# Gray-white matter boundary Z-score and volume as imaging biomarkers of Alzheimer’s disease

**DOI:** 10.3389/fnagi.2023.1291376

**Published:** 2023-12-14

**Authors:** Yunan Tian, Jang-Hoon Oh, Hak Young Rhee, Soonchan Park, Chang-Woo Ryu, Ah Rang Cho, Geon-Ho Jahng

**Affiliations:** ^1^Department of Medicine, Graduate School, Kyung Hee University College of Medicine, Seoul, Republic of Korea; ^2^Department of Radiology, Kyung Hee University Hospital, Kyung Hee University College of Medicine, Seoul, Republic of Korea; ^3^Department of Neurology, Kyung Hee University Hospital at Gangdong, Kyung Hee University College of Medicine, Seoul, Republic of Korea; ^4^Department of Radiology, Kyung Hee University Hospital at Gangdong, Kyung Hee University College of Medicine, Seoul, Republic of Korea; ^5^Department of Psychiatry, Kyung Hee University Hospital at Gangdong, Kyung Hee University College of Medicine, Seoul, Republic of Korea

**Keywords:** Alzheimer’s disease, gray-white matter boundary Z-score, boundary tissue volume, cognitive impairment, age

## Abstract

**Introduction:**

Alzheimer’s disease (AD) presents typically gray matter atrophy and white matter abnormalities in neuroimaging, suggesting that the gray-white matter boundary could be altered in individuals with AD. The purpose of this study was to explore differences of gray-white matter boundary Z-score (gwBZ) and its tissue volume (gwBTV) between patients with AD, amnestic mild cognitive impairment (MCI), and cognitively normal (CN) elderly participants.

**Methods:**

Three-dimensional T1-weight images of a total of 227 participants were prospectively obtained from our institute from 2006 to 2022 to map gwBZ and gwBTV on images. Statistical analyses of gwBZ and gwBTV were performed to compare the three groups (AD, MCI, CN), to assess their correlations with age and Korean version of the Mini-Mental State Examination (K-MMSE), and to evaluate their effects on AD classification in the hippocampus.

**Results:**

This study included 62 CN participants (71.8 ± 4.8 years, 20 males, 42 females), 72 MCI participants (72.6 ± 5.1 years, 23 males, 49 females), and 93 AD participants (73.6 ± 7.7 years, 22 males, 71 females). The AD group had lower gwBZ and gwBTV than CN and MCI groups. K-MMSE showed positive correlations with gwBZ and gwBTV whereas age showed negative correlations with gwBZ and gwBTV. The combination of gwBZ or gwBTV with K-MMSE had a high accuracy in classifying AD from CN in the hippocampus with an area under curve (AUC) value of 0.972 for both.

**Conclusion:**

gwBZ and gwBTV were reduced in AD. They were correlated with cognitive function and age. Moreover, gwBZ or gwBTV combined with K-MMSE had a high accuracy in differentiating AD from CN in the hippocampus. These findings suggest that evaluating gwBZ and gwBTV in AD brain could be a useful tool for monitoring AD progression and diagnosis.

## Introduction

Alzheimer’s disease (AD) is a neurodegenerative disease that causes progressive memory loss and cognitive impairment. AD is characterized by accumulation of β-amyloid (Aβ) plaques and hyperphosphorylated tau protein in the brain, which can impair neuronal function and communication (Selkoe, 1993; Augustinack et al., 2002). Mild cognitive impairment (MCI) is a condition that precedes AD, in which individuals experience episodic memory loss more than normal aging, but not enough to meet the criteria for AD (Petersen et al., 1999, 2001). Early detection and intervention of MCI might prevent and/or delay the onset of AD, thus improving the quality of life of patients and caregivers. However, finding reliable biomarkers for MCI and AD remains a challenge.

One of the main challenges in AD research is to identify reliable biomarkers that can detect the disease at an early stage and monitor its progression. Neuroimaging techniques such as positron emission tomography (PET) and magnetic resonance imaging (MRI) have been widely used to study brain changes associated with AD. MRI can assess structural and functional alterations in gray matter (GM) and white matter (WM), which reflect neurodegeneration and connectivity loss in MCI and AD (Phillips et al., 2016; Minkova et al., 2017). However, most neuroimaging studies have focused on either GM or WM separately, neglecting possible interactions between them.

The interface between GM and WM is called gray-white matter boundary (gwB), a region that contains abundant neural cell bodies and myelinated axons. A recent study has suggested that gwB is vulnerable to axonal injury and stress, which might contribute to neurocognitive dysfunction. This widespread axonal damage is secondary to abnormal accumulation of amyloid precursor protein (APP) which is closely correlated with AD (Alisafaei et al., 2020). Neural cells sense and respond to changes in the physical properties of their surroundings by adjusting their cytoskeletal structure and this kind of alteration and disruption of cytoskeletal structure is closely related to neurodegenerative diseases (Smith and Meaney, 2000). Moreover, the histopathological structure of gwB integrity has been found to be associated with memory performance in healthy older adults (Blackmon et al., 2019). Therefore, assessing gwB might provide novel insights into neural mechanisms of MCI and AD.

Neurodegeneration is mainly related to the loss and migration of neurons and myelin damage, which lead to structural damage (West et al., 1994; Guo et al., 2012; Dean et al., 2017). The gray-white matter boundary serves as the junction between neuron cell bodies and axons, making it easier to observe related changes in neurons and myelin at the same time. Previous study suggested that alteration of gray-white matter boundary could indicate local variations in myelin degradation (Koenig, 1991). Moreover, poor differentiation of the gray–white matter boundary along with supernumerary neurons in the subcortical white matter was closely related to neurodegeneration disease (Simms et al., 2009; Avino and Hutsler, 2010).

Since previous studies have shown a link between neurocognitive dysfunction and the gray-white matter interface (Blackmon et al., 2019; Alisafaei et al., 2020), the aim of this study was to investigate differences in gwB and its tissue volumes among three groups of participants: cognitively normal (CN) elderly, amnestic MCI, and AD. We also examined relationships of gwB and its tissue volume (gwBTV) with neurocognitive function as well as the diagnostic accuracy of gwB for differentiating AD from others. We hypothesized that gwB would be more blurred in MCI and AD than in CN and that gwB blurring would correlate with cognitive impairment and brain atrophy. We also expected that gwB would be a useful biomarker for identifying AD patients.

## Methods and materials

### Participants

Participants were enrolled in several prospective studies conducted at our institute between 2006 and 2022. All studies were approved by the Institutional Review Board (KHNMC IRB 2007–005, KHNMC IRB 2009–056, KHNMC IRB 2011–059, KHNMC IRB 2015–02-006 and KHNMC IRB 2019–07-007). All participants gave informed consent. Participants who underwent neurologic examination, neuropsychological testing using the Seoul Neuropsychological Screening Battery (SNSB), which included the Korean version of the Mini-Mental State Examination (K-MMSE) (Ahn et al., 2010) and MRI, were recruited from our neurology center. Two experienced neuroradiologists reviewed brain images of each participant and excluded those with prior cortical infarcts or other space-occupied lesions.

**Figure 1:**
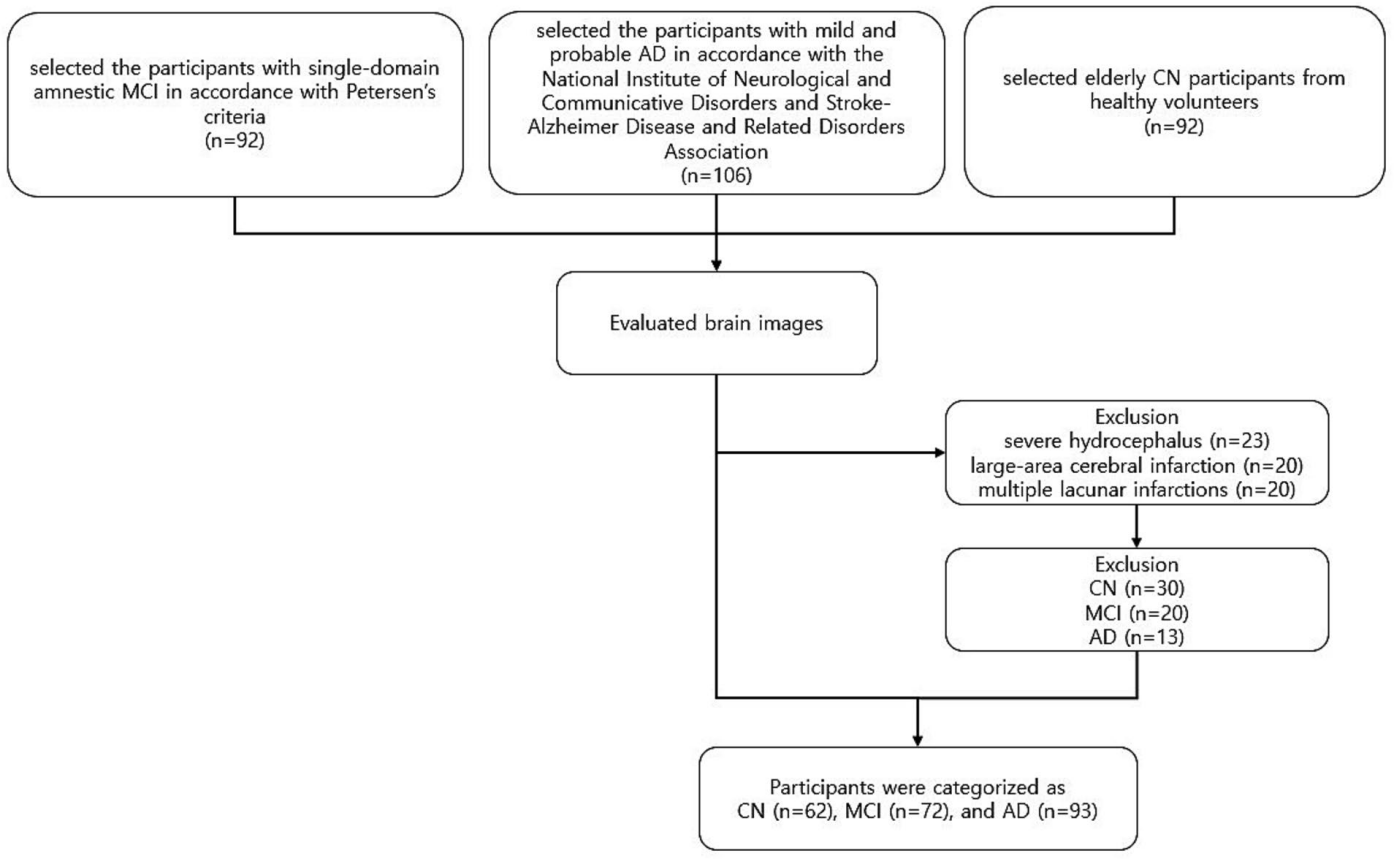
Overview of exclusion and inclusion criteria used for the selection process of participants. Participants were enrolled in several prospective studies conducted at our institute between 2006 and 2022. Participants who underwent neurologic examination, neuropsychological testing, and MRI, were recruited from our neurology center. Two experienced neuroradiologists reviewed brain images of each participant and excluded those with prior cortical infarcts or other space-occupied lesions. The final 227 sample consisted of 62 CN, 72 MCI, and 93 AD participants. CN, cognitively normal; MCI, mild cognitive impairment; AD, Alzheimer’s disease.

We selected participants with single-domain amnestic MCI according to Petersen’s criteria (Petersen et al., 1999, 2001), which included: (1) cognitive complaints by the patient or caregiver; (2) normal general cognitive function on the K-MMSE (K-MMSE≥24); (3) cognitive impairment on objective testing; (4) normal activities of daily living; and (5) no dementia. Cognitive impairment with no dementia is defined as an individual whose cognitive function is at a level below normal and clinical dementia rating (CDR) =0.5, but for whom dementia symptoms are not evident or even do not meet criteria for dementia (Tuokko et al., 2001). We also selected patients with mild and probable AD with CDR scores of 0.5, 1, or 2 based on the criteria of the National Institute of Neurological and Communicative Disorders and Stroke-Alzheimer Disease and Related Disorders Association (Mckhaann et al., 1984), which included: (1) dementia confirmed by clinical examination and neuropsychological tests; (2) deficits in two or more areas of cognition; (3) progressive worsening of memory and other cognitive functions; (4) no disturbance of consciousness; (5) onset age between 40 and 90 years of age; and (6) absence of other systemic or neurologic disorders that could explain cognitive defects. Elderly CN participants were healthy volunteers without a history of neurological disease and with a normal brain MRI means that brain does not have any abnormalities in the brain such as brain tumors, cortical infarcts, or other space-occupied lesions on MR images. We selected 290 participants from the database of the five projects for this study. We excluded 63 participants (30 CN, 20 MCI, and 13 AD) who had severe hydrocephalus (*N* = 23), large-area cerebral infarction (*N* = 20), or multiple lacunar infarctions (*N* = 20). The final 227 sample consisted of 62 CN, 72 MCI, and 93 AD participants (Figure 1). Table 1 shows their demographic characteristics and neuropsychologic test results.

**Table 1:** Summary of group comparisons of demographic data and neuropsychologic test of participants.

Group	CN (1)	MCI (2)	AD (3)	*p* value (post-hoc)
Participants	62	72	93	227 (total)
*Age (years)	71.8 ± 4.8	72.6 ± 5.1	73.6 ± 7.7	F = 1.461, *p* = 0.225
^#^Sex (M/F)	20/42 (32.3%/67.7%)	23/49 (31.9%/68.1%)	22/71 (23.7/76.3)	χ^2^ ≤ 0.593, *p* > 0.441
*TIV (cm^3^)	1.354 ± 0.112	1.356 ± 0.112	1.342 ± 0.112	F = 0.329, P = 0.720
*K-MMSE	27.4 ± 2.5	25.8 ± 3.1	19.4 ± 4.1	F = 121.335, P < 0.001 (*p* = 0.0017, 1,2), (*p* < 0.0001, 1,3), (*p* < 0.0001, 2,3)
CDR	0(0–0.5)	0.5(0.5)	1(0.5–1.0)	N/A

*Value of *p* by one-way analysis of variance (ANOVA) with Scheffé test for the post hoc test.

#*p*-value by the chi-squared test.

Data of age, K-MMSE, and TIV are presented as mean ± standard deviation. CDRs are presented as median (range). Results of the post hoc test are described as follows: significant difference among the three groups of subjects as (1,2,3), between CN and AD as (1,3), between CN and MCI as (1,2), and between MCI and AD as (2,3).

K-MMSE, Korean version of the Mini-Mental State Examination; TIV, total intracranial volume; CDR, Clinical Dementia Rate; AD, Alzheimer’s disease; MCI, Mild cognitive impairment; CN, cognitively normal.

### MRI acquisition

We used a 3-T MR system (Achieva or Ingenia, Philips Medical Systems, Best, The Netherlands) to perform MRI scans for each participant. We acquired sagittal structural 3D T1WI using a turbo field echo sequence with these imaging parameters: repetition time (TR) = 8.1 ms, echo time (TE) = 3.7 ms, flip angle (FA) = 8°, field-of-view (FOV) = 236 × 236 mm^2^, and acquisition voxel size = 1 × 1 × 1 mm^3^. We also acquired T2-weighted turbo-spin-echo and fluid-attenuated inversion recovery (FLAIR) images to check the brain for any abnormalities. We reviewed all scans before processing to ensure the absence of motion and other artifacts.

### Pre-processing of MRI images

**Figure 2:**
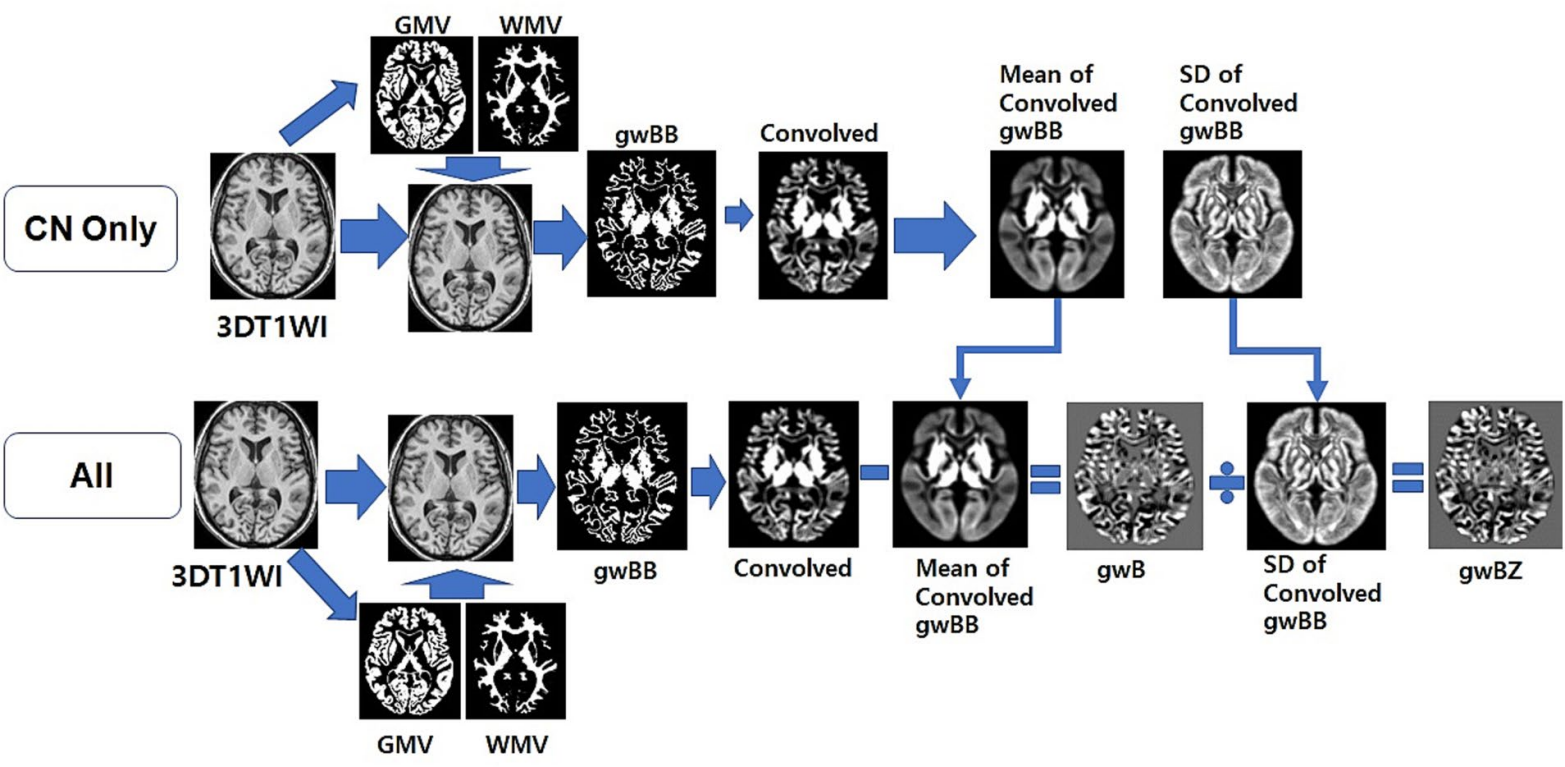
Overview of the optimized processing pipeline to map gray-white matter boundary binary (gwBB) and gray-white matter boundary Z-score (gwBZ). We first normalized the 3D T1WI of each participant to our AD brain template, and segmented into GM, WM, cerebrospinal fluid (CSF), and others using SPM12. Second, we created a binary map with intensity value of normalized 3D T1WI and location information of segmented GM and WM brain tissues. Third, we set lower and upper thresholds of the binary calculation using the equation shown in the text and information of both signal intensities and brain tissue volumes. Fourth, we generated a convolved map by applying a 3D convolution filter. Fifth, we created an individual gray-white matter boundary (gwB) map by subtracting the individual convolved map from the mean convolved map. Finally, we created a gray-white matter boundary Z-score (gwBZ) map by dividing the gwB map by the SD convolved map. CN, cognitively normal; 3DT1WI, three dimensional T1 weighted image; GMV, gray matter volume; WMV, white matter volume; gwB, gray-white matter boundary; SD, standard deviation.

We used local MATLAB programming with Statistical Parametric Mapping version 12 (SPM12) software (Welcome Department of Imaging Neuroscience, University College, London, UK) to calculate gwB. We followed the method for constructing gwB proposed in a previous study (Huppertz et al., 2008) and optimized the processing pipeline to map gwB (Figure 2). First, we wrapped the 3D T1WI of each participant to our AD brain template (Guo et al., 2021) to normalize the brain and to segment brain tissues into GM, WM, cerebrospinal fluid (CSF), and others using SPM12. Total intracranial volume (TIV) was obtained by summation of all brain tissues in the voxel after segmenting the brain. Second, we created a binary map with intensity value of normalized 3D T1WI and location information of segmented GM and WM brain tissues. We used the following equations (Huppertz et al., 2008) to set lower and upper thresholds of the binary calculation:


Thresholdlower=SIGM50Mean+12SIGM502SD

and


Thresholdupper=SIWM50Mean−12SIWM502SD

where $SI_{GM50}^{\mathrm{Mean}}$ and $SI_{WM50}^{\mathrm{Mean}}$ were average signal intensities over more than 50% volume of gray matter and 50% volume of white matter in the voxel, respectively. $SI_{GM50}^{2SD}$ and $SI_{WM50}^{2SD}$ were dispersion of the signal intensity defined by two standard deviation (SD) in more than 50% gray matter volume and white matter volume, respectively. Lower and upper thresholds were determined by mean and SD over partial volume percentage of GM and WM volumes. We assigned 1 to threshold range and 0 to the rest. Thus, the binary map set thresholds to 1 or 0 called as gray-white matter binary boundary (gwBB) map, which is just like to line that some voxels have no values, differs the line shape among the participants, and does not have information of distribution, and is nosing. Therefore, the gwBB map needs to blur some levels to minimize the variation among the participants, can denoise the image, and make the calculation of z-scores more efficient. Third, we generated a convolved map by applying a 3D convolution filter with a cubic matrix of 5 × 5 × 5 to the gwBB map. We used the 5x5x5 cubic matrix of 3D convolution filter in our study decided by our imaging processing experiment. At this point, the convolved gwBB maps from all control CN participants were averaged to calculate mean maps of the convolved gwBB and calculated to SD maps of the convolved gwBB using a mathlab code. Those mean and SD maps were used to calculate a gray-white matter boundary Z-score (gwBZ) map of all participants. Fourth, we created an individual gwB map by subtracting the individual convolved gwBB map from the mean convolved gwBB map and then divided it by SD convolved gwBB map to calculate gwBZ map for each participant. Finally, we obtained gwBTV, which was the boundary tissue volume at the area of gwBB. Before performing voxel-based statistical analyses, we applied Gaussian smoothing with an 8 mm isotropic full-width at half maximum (FWHM) to all maps.

### Statistical analyses

*Demographic data and neuropsychological test:* To compare age, total TIV, and K-MMSE score among the three participant groups, a one-way analysis of variance (ANOVA) was used. Scheffé’s post-hoc tests were performed for pairwise comparisons of subgroups when significant differences were found. Chi-square test was used to compare gender proportions between groups.

*Voxel-based analysis:* Statistical analyses of gwBZ and gwBTV maps were performed using the voxel-based method. First, voxel-wise full factorial one-way analysis of covariance (ANCOVA) was used to compare each map among groups with age and TIV as covariates. Second, the voxel-based multiple regression analysis was used to assess the association between each map and either age with TIV as a covariate or K-MMSE score with both age and TIV as covariates. False-discovery rate (FDR) method was applied for multiple comparisons with a significance level of α = 0.01 and clusters with at least 100 contiguous voxels.

*Region-of-interest (ROI)-based analysis*: ROIs were defined in three ways based on brain function areas and voxel-based analysis results. First, ROIs were defined at lobes of the brain such as frontal, parietal, occipital, temporal, and limbic lobes. Second, ROIs were defined based on voxel-based analysis results at parahippocampal, thalamus, middle temporal gyrus (MTG), insula, precuneus, and cuneus. Finally, ROIs were defined in areas related to AD such as the hippocampus and neocortex (Brodmann area 24, 25, 30). The WFU_PickAtlas software toolbox (fmri.wfubmc.edu/ software/pickatlas) was used to map these regions. Average values of gwBZ and gwBTV from all ROIs were obtained using Marsbar software (Matthew Brett, http://marsbar.sourceforge.net).

The following statistical analyses were performed using ROI values. First, ANCOVA was used to compare gwBZ or gwBTV values among the three participant groups for each ROI with age and TIV as covariates. Bonferroni correction as the post-hoc test was performed when a significant difference between groups was found. Second, partial correlation analysis was performed for all participants to evaluate the relationship between gwBZ or gwBTV values and K-MMSE scores for each ROI adjusted by participant’s age and TIV. Third, Pearson correlation analyses were performed to determine relationships between ROI values and age for all participants and for each participant group. Finally, logistic regression analysis was performed on gwBZ or gwBTV in combination with K-MMSE scores and save the predicted probabilities in each ROI. The predicted value was used to analyze classification of three groups by receiver operating characteristic (ROC) curve analysis. A significance level of α = 0.05 was applied for all ROI analyses. The MedCalc statistical software (http://www.medcalc.org/, Ostend, Belgium) was used to perform all statistical analyses for ROI values.

## Results

### Participants characteristics

Table 1 summarizes demographic data and neuropsychological test results of participants. Overall, 62 CN (71.8 ± 4.8 years, 20 males and 42 females), 72 MCI (72.6 ± 5.1 years, 23 males and 49 females), and 93 AD (73.6 ± 7.7 years, 22 males and 71 females) elderly were included. There was no significant differences in age (*F* = 1.461, *p* = 0.225), sex (χ^2^ ≤ 0.593, *p* > 0.441), or TIV (*F* = 0.329, *p* = 0.720) among CN, MCI, and AD groups. As expected, K-MMSE was significantly different among the three groups as indicated by ANOVA (*F* = 121.335, *p* < 0.001). Post-hoc test showed that K-MMSE values were significantly lower in AD than in CN or MCI group and in MCI than in CN group.

**Figure 3:**
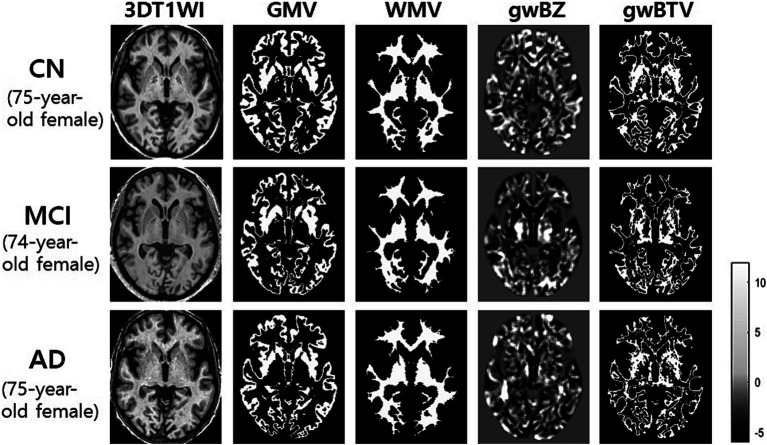
Representative maps of gray-white matter boundary Z-score (gwBZ) and its boundary tissue volume (gwBTV) maps obtained from three participants who were females with a similar age. We obtained those maps from one participant (75-year-old female) with cognitively normal (CN) elderly, one patient (74-year-old female) with mild cognitive impairment (MCI), and one patient (75-year-old female) with Alzheimer’s disease (AD). The color bar indicates the specific value of Z-score. CN, cognitively normal; MCI, mild cognitive impairment; AD, Alzheimer’s disease; 3DT1WI, three dimensional T1 weight image; GMV, gray matter volume; WMV, white matter volume.

Figure 3 shows representative maps of gwBZ and gwBTV obtained from one CN participant (a 75-year-old), one MCI participant (a 74-year-old), and one AD participant (a 75-year-old) who were females with a similar age. Bright regions in the boundary map indicate cortical areas and the transition zone between gray and white matter. Compared to CN and MCI, gwBZ of AD had less bright regions, especially in the bilateral occipital lobe, hippocampus area, and insula.

### Voxel-based analyses

**Figure 4:**
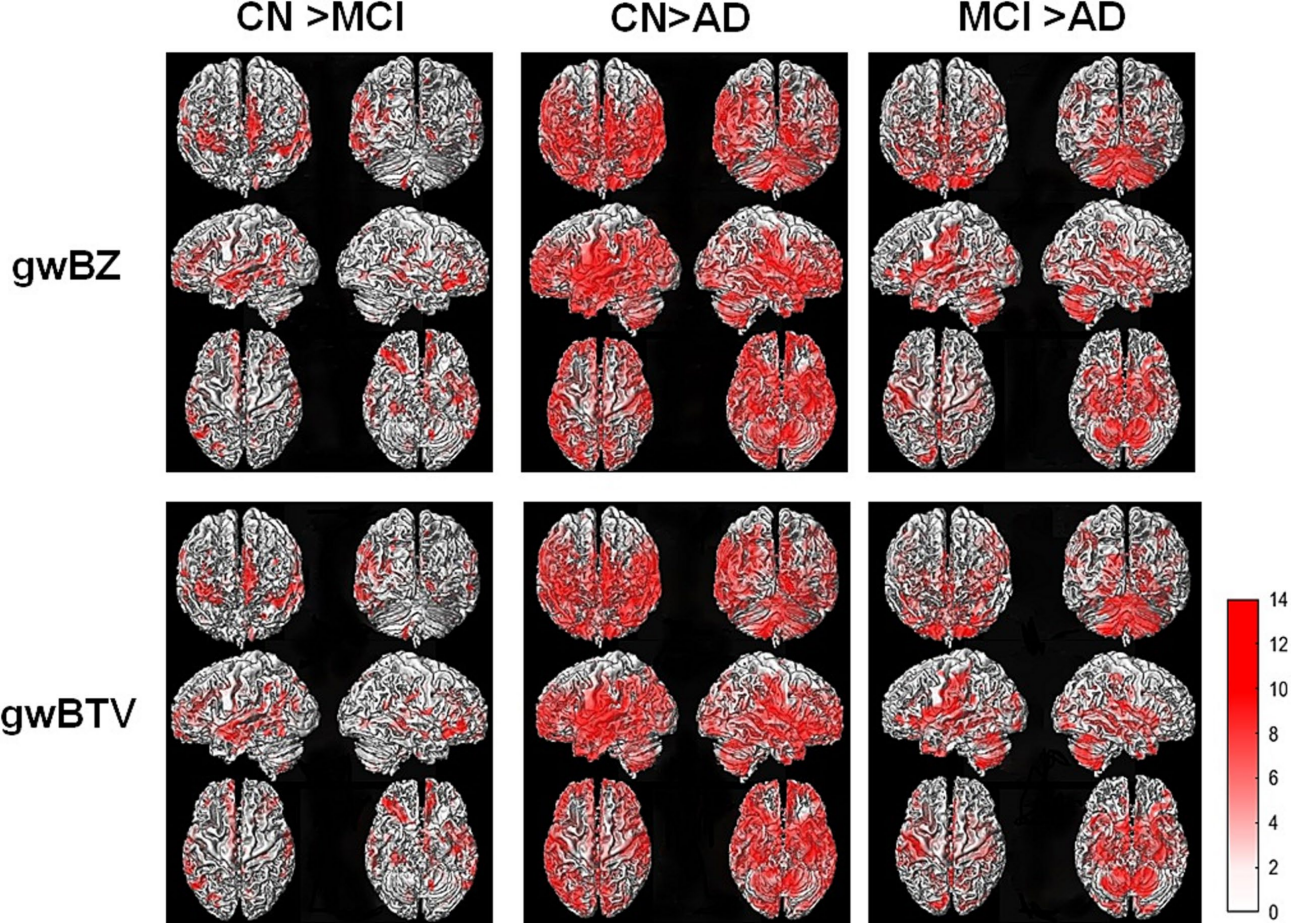
Results of voxel-based analysis of covariance (ANCOVA) of gray-white matter boundary Z-score (gwBZ) and gray-white matter boundary tissue volume (gwBTV) among the three participant groups (CN, MCI, and AD). Results are overlaid to the rendered gray-white matter boundary. Red color indicates areas where gwBZ and gwBTV are significantly different between the groups. Statistical significance was reached if *p* < 0.01 and false discovery rate corrected for the multiple comparison. The color bar presents the T-value under significant difference areas. Supplementary Figure S1 shows the result overlaid into the axial slices of the gray-white matter boundary map. CN, cognitively normal; MCI, mild cognitive impairment; AD, Alzheimer’s disease; gwBZ, gray-white matter boundary Z-score; gwBTV, gray-white matter boundary tissue volume.

*Group comparison of gwBZ and gwBTV maps*: Figure 4 shows voxel-based comparison results of gwBZ maps and gwBTV maps among participant groups. It was found that gwBZ and gwBTV were lower in AD than in CN or MCI and lower in MCI than in CN. Since Figure 4 shows the results overlapped in the 3-dimensional rendered image of gwBB, we additionally supplied Supplementary Figure S1, which shows the same result, but in the axial slices of gwBB for each comparison.

**Figure 5:**
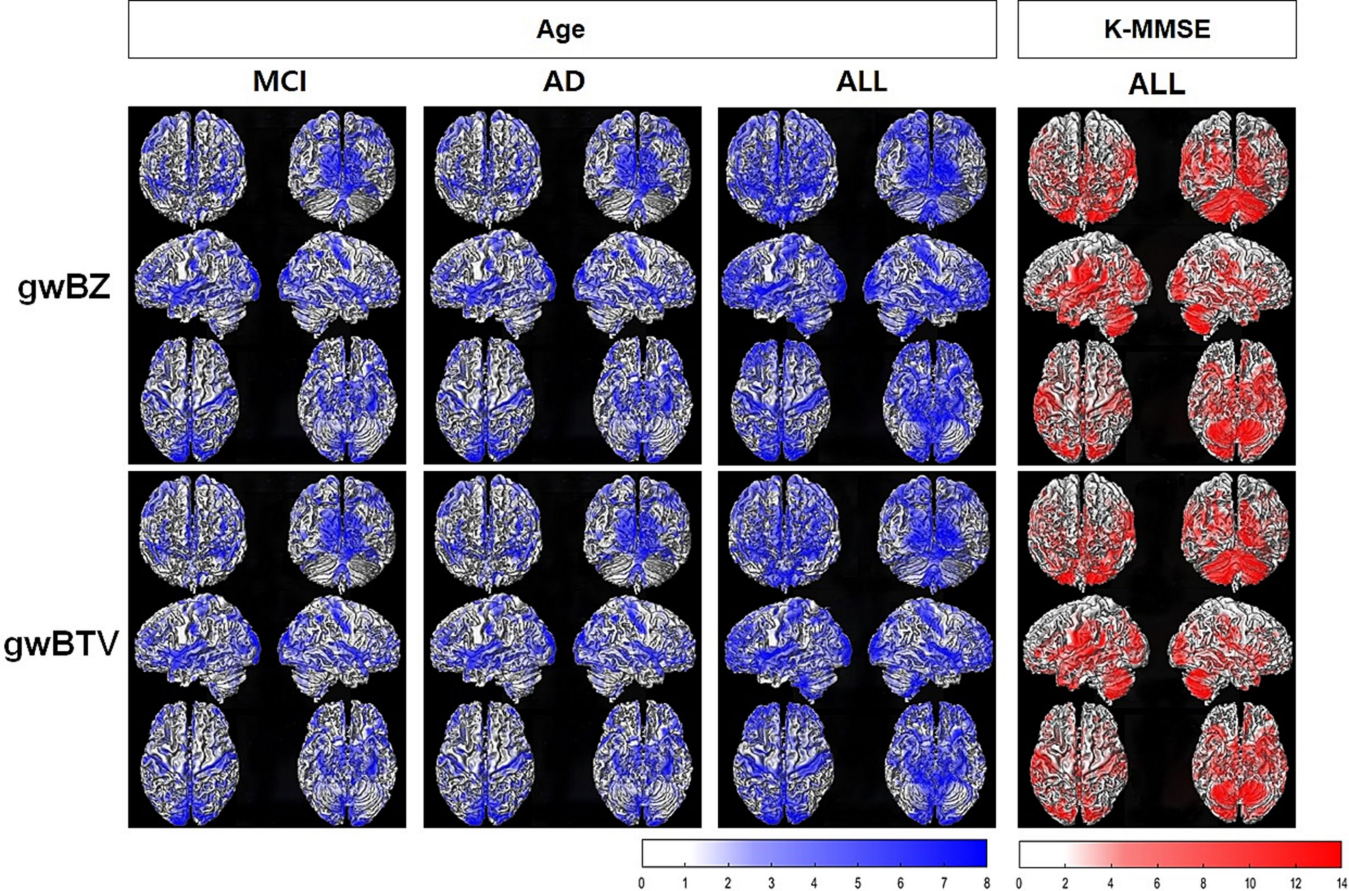
Results of voxel-based multiple regression analysis of gray-white matter boundary Z-score (gwBZ) and gray-white matter boundary tissue volume (gwBTV) maps with Korean version of the Mini-Mental State Examination (K-MMSE) or age in each participant group and/or all participants. Results are overlaid to the rendered gray-white matter boundary. Blue color indicates areas where gwBZ or gwBTV is negatively correlated with age, and red color indicates areas where gwBZ or gwBTV is positively correlated with K-MMSE. Statistical significance was reached if p < 0.01 and false discovery rate corrected for the multiple comparison. The color bar presents the T-value under significant difference areas. In addition, Supplementary Figure S2, S3 show the results of gwBZ and gwBTV overlaid into the axial slices of the gray-white matter boundary map, respectively. CN, cognitively normal; MCI, mild cognitive impairment; AD, Alzheimer’s disease; K-MMSE, Korean version of the Mini-Mental State Examination.

*Association of gwBZ and gwBTV maps with age and K-MMSE:* Figure 5 shows voxel-based multiple regression results of gwBZ and gwBTV maps with age or K-MMSE. Age had negative associations with both gwBZ and gwBTV in MCI, AD, and all participants. K-MMSE had positive associations with both gwBZ and gwBTV in all participants, but not in each group. Since Figure 5 shows the results overlapped in the 3-dimensional rendered image of gwBB, we additionally supplied Supplementary Figure S2 for the correlation of gwBZ and Supplementary Figure S3 for the correlation of gwBTV, which show the same results, but in the axial slices of gwBB.

### ROI-based analyses

*Group comparison of ROI values:* Table 2 lists results of comparison between gwBZ and gwBTV among the three groups in each ROI. Both gwBZ and gwBTV showed statistically significant differences for all ROIs among the three groups. The Bonferroni-corrected post-hoc analysis showed that gwBZ values were significantly lower in AD than in CN and MCI in all ROIs except precuneus and the frontal lobe. Compared to CN, MCI had significantly lower gwBZ in neocortex, MTG, parahippocampus, thalamus, limbic lobe, and temporal lobe. Negative values of gwBZ indicate that the gwBZ value for each participant is smaller than that of the mean population. Moreover, gwBTV values were significantly lower in AD than in CN and MCI at all ROIs except MTG, precuneus, the frontal lobe, and the parietal lobe. Compared to CN, MCI had significantly lower gwBTV in the hippocampus, neocortex, MTG, thalamus, limbic lobe, and temporal lobe.

**Table 2:** Results of comparing gray-white matter boundary Z-score (gwBZ) and gray-white boundary tissue volume (gwBTV) values among the three participant groups in specific brain areas.

ROI	CN (1) (mean ± SD)	MCI (2) (mean ± SD)	AD (3) (mean ± SD)	ANCOVA (*F, p*)	*Post hoc* (Bonferroni corrected, p*)
gwBZ					
Hippocampus	−0.213 ± 0.303	−0.324 ± 0.349	−0.574 ± 0.344	*F* = 31.408 *p* < 0.001	(*p* < 0.001, 1,3) (*p* < 0.001, 2,3)
Neocortex	−0.113 ± 0.174	−0.177 ± 0.157	−0.286 ± 0.159	*F* = 30.611 *p* < 0.001	(*p* = 0.023, 1,2) (*p* < 0.001, 1,3) (*p* < 0.001, 2,3)
Cuneus	−0.091 ± 0.227	−0.116 ± 0.210	−0.205 ± 0.220	*F* = 7.559 *p* = 0.001	(*p* = 0.001, 1,3) (*p* = 0.012, 2,3)
Insula	−0.143 ± 0.190	−0.198 ± 0.165	−0.336 ± 0.191	*F* = 33.284 *p* < 0.001	(*p* < 0.001, 1,3) (*p* < 0.001, 2,3)
MTG	−0.032 ± 0.124	−0.105 ± 0.117	−0.144 ± 0.125	*F* = 15.801 *p* < 0.001	(*p* = 0.002, 1,2) (*p* < 0.001, 1,3)
Parahippocampal	−0.165 ± 0.220	−0.242 ± 0.250	−0.381 ± 0.252	*F* = 19.468 *p* < 0.001	(*p* < 0.001, 1,2) (*p* < 0.001, 2,3)
Precuneus	−0.040 ± 0.144	−0.088 ± 0.142	−0.122 ± 0.138	*F* = 6.948 *p* = 0.001	(*p* < 0.001, 1,3)
Thalamus	−0.251 ± 0.356	−0.410 ± 0.415	−0.720 ± 0.418	*F* = 44.193 *p* < 0.001	(*p* = 0.012, 1,2) (*p* < 0.001, 1,3) (*p* < 0.001, 2,3)
Frontal lobe	−0.049 ± 0.114	−0.081 ± 0.098	−0.106 ± 0.101	*F* = 5.987 *p* < 0.003	(*p* = 0.002, 1,3)
Limbic lobe	−0.104 ± 0.158	−0.177 ± 0.155	−0.252 ± 0.158	*F* = 21.536 *p* < 0.001	(*p* = 0.007, 1,2) (*p* < 0.001, 1,3) (*p* = 0.002, 2,3)
Occipital lobe	−0.069 ± 0.158	−0.085 ± 0.156	−0.166 ± 0.156	*F* = 10.577 *p* < 0.001	(*p* < 0.001, 1,3) (*p* = 0.001, 2,3)
Parietal lobe	−0.036 ± 0.133	−0.067 ± 0.133	−0.115 ± 0.114	*F* = 8.240 *p* < 0.001	(*p* < 0.001, 1,3) (*p* = 0.040, 2,3)
Temporal lobe	−0.054 ± 0.115	−0.127 ± 0.113	−0.197 ± 0.106	*F* = 33.078 *p* < 0.001	(*p* < 0.001, 1,2) (*p* < 0.001, 1,3) (*p* < 0.001, 2,3)
gwBTV					
Hippocampus	0.229 ± 0.036	0.216 ± 0.041	0.187 ± 0.041	*F* = 31.391 *p* < 0.001	(*p* < 0.001, 1,2) (*p* < 0.001, 1,3) (*p* < 0.001, 2,3)
Neocortex	0.224 ± 0.020	0.217 ± 0.018	0.203 ± 0.019	*F* = 33.913 *p* < 0.001	(*p* = 0.030, 1,2) (p < 0.001, 1,3) (p < 0.001, 2,3)
Cuneus	0.267 ± 0.030	0.267 ± 0.028	0.254 ± 0.029	*F* = 7.878 *p* < 0.001	(p = 0.001, 1,3) (*p* = 0.008, 2,3)
Insula	0.229 ± 0.024	0.222 ± 0.021	0.205 ± 0.024	*F* = 30.964 *p* < 0.001	(p < 0.001, 1,3) (p < 0.001, 2,3)
MTG	0.186 ± 0.016	0.176 ± 0.015	0.171 ± 0.016	*F* = 16.820 *p* < 0.001	(p < 0.001, 1,2) (*p* < 0.001, 1,3)
Parahippocampal	0.247 ± 0.027	0.238 ± 0.030	0.220 ± 0.031	*F* = 20.178 *p* < 0.001	(*p* < 0.001, 1,3) (*p* < 0.001, 2,3)
Precuneus	0.218 ± 0.020	0.211 ± 0.019	0.206 ± 0.019	*F* = 7.479 *p* = 0.001	(*p* < 0.001, 1,3)
Thalamus	0.547 ± 0.052	0.524 ± 0.061	0.480 ± 0.061	*F* = 42.320 *p* < 0.001	(*p* = 0.016, 1,2) (*p* < 0.001, 1,3), (*p* < 0.001, 2,3)
Frontal lobe	0.186 ± 0.015	0.181 ± 0.013	0.178 ± 0.014	*F* = 6.641 *p* = 0.002	(*p* = 0.001, 1,3)
Limbic lobe	0.209 ± 0.019	0.201 ± 0.018	0.191 ± 0.019	*F* = 22.367 *p* < 0.001	(*p* = 0.007, 1,2) (*p* < 0.001, 1,3) (*p* = 0.001, 2,3)
Occipital lobe	0.225 ± 0.020	0.222 ± 0.019	0.212 ± 0.019	*F* = 11.645 *p* < 0.001	(*p* < 0.001, 1,3) (*p* < 0.001, 2,3)
Parietal lobe	0.199 ± 0.019	0.194 ± 0.018	0.190 ± 0.016	*F* = 7.906 *p* < 0.001	(*p* < 0.001, 1,3)
Temporal lobe	0.195 ± 0.015	0.186 ± 0.014	0.177 ± 0.014	*F* = 32.542 *p* < 0.001	(*p* < 0.001, 1,2), (*p* < 0.001, 2,3) (*p* < 0.001, 1,3)

p* by one-way analysis of variance (ANCOVA) with Bonferroni corrected post hoc test.

Data of gwBZ and gwBTV for each group are presented as mean ± standard deviation. Results of the post hoc test are described as follows: significant difference between CN and AD as (1,3), between CN and MCI as (1,2), and between MCI and AD as (2,3).

ROI, regions-of-interest; CN, cognitively normal; MCI, mild cognitive impairment; AD, Alzheimer’s disease; gwBZ, gray-white matter boundary Z-score; gwBTV, gray-white matter boundary tissue volume, MTG, middle temporal gyrus, SD, standard deviation.

*Correlation of gwBZ and gwBTV with K-MMSE and age:* Table 3 lists results of the correlation analysis of gwBZ and gwBTV with age in each ROI and results of the partial correlation analysis of those values with K-MMSE adjusted by age and TIV. Both gwBZ and gwBTV had negative correlations with age in all ROIs. Supplementary Table S1 shows additional results for other ROIs. Also, both gwBZ and gwBTV had negative correlations with age for each group in all ROIs. In addition, both gwBZ and gwBTV had positive correlations with the K-MMSE in all ROIs. To enhance the presentation of the results in Table 3, we added Supplementary Figure S5 to present the results of the correlation of gwBZ or gwBTV with age or K-MMSE.

**Table 3:** Results of correlation analysis of gray-white matter boundary Z-score (gwBZ) and gray-white matter boundary tissue volume (gwBTV) with age or K-MMSE in specific brain areas using all participant data.

ROI	Map	Age (r, *p*)	*K-MMSE (r, *p*)
Hippocampus	gwBZ	r = −0.413, *p* < 0.001	r = 0.312, *p* < 0.001
	gwBTV	r = −0.415, *p* < 0.001	r = 0.323, *p* < 0.001
Neocortex	gwBZ	r = −0.384, *p* < 0.001	r = 0.292, *p* < 0.001
	gwBTV	r = −0.383, *p* < 0.001	r = 0.315, *p* < 0.001
Cuneus	gwBZ	r = −0.457, *p* < 0.001	r = 0.251, *p* < 0.001
	gwBTV	r = −0.463, *p* < 0.001	r = 0.266, *p* < 0.001
Insula	gwBZ	r = −0.403, *p* < 0.001	r = 0.312, *p* < 0.001
	gwBTV	r = −0.412, *p* < 0.001	r = 0.295, *p* < 0.001
MTG	gwBZ	r = −0.120, *p* = 0.073	r = 0.225, *p* < 0.001
	gwBTV	r = −0.104, *p* = 0.120	r = 0.226, *p* < 0.001
Parahippocampal	gwBZ	r = −0.356, *p* < 0.001	r = 0.233, *p* < 0.001
	gwBTV	r = −0.345, *p* < 0.001	r = 0.235, *p* < 0.001
Precuneus	gwBZ	r = −0.313, *p* < 0.001	r = 0.188, *p* < 0.001
	gwBTV	r = −0.315, *p* < 0.001	r = 0.200, *p* = 0.003
Thalamus	gwBZ	r = −0.421, *p* < 0.001	r = 0.373, *p* < 0.001
	gwBTV	r = −0.422, *p* < 0.001	r = 0.369, *p* < 0.001
Frontal lobe	gwBZ	r = −0.309, *p* < 0.001	r = 0.107, *p* = 0.108
	gwBTV	r = −0.327, *p* < 0.001	r = 0.112, *p* = 0.094
Limbic lobe	gwBZ	r = −0.366, *p* < 0.001	r = 0.225, *p* < 0.001
	gwBTV	r = −0.342, *p* < 0.001	r = 0.233, *p* < 0.001
Occipital lobe	gwBZ	r = −0.411, *p* < 0.001	r = 0.260, *p* < 0.001
	gwBTV	r = −0.404, *p* < 0.001	r = 0.274, *p* < 0.001
Parietal lobe	gwBZ	r = −0.293, *p* < 0.001	r = 0.220, *p* < 0.001
	gwBTV	r = −0.302, *p* < 0.001	r = 0.206, *p* = 0.002
Temporal lobe	gwBZ	r = −0.266, *p* < 0.001	r = 0.320, *p* < 0.001
	gwBTV	r = −0.251, *p* < 0.001	r = 0.311, *p* < 0.001

Results of correlation analysis for age are listed as correlation coefficient (r) and *p*-value. *Results of partial correlation for K-MMSE score with covariates as participant’s age and total intercranial volume (TIV) are listed as correlation coefficient (r) and value of *p*.

gwBZ, gray-white matter boundary Z-score; gwBTV, gray-white matter boundary tissue volume; ROI, regions-of-interest; MTG, middle temporal gyrus; K-MMSE, Korean version of the Mini-Mental State Examination.

*ROC curve analysis*: Table 4 summarizes results of ROC curve analysis of gwBZ and gwBTV values for group classification in the hippocampus. For classifying AD from CN, AUC values of gwBZ and gwBTV were 0.798 and 0.795, respectively. When gwBZ and gwBTV were combined, the AUC value increased slightly (0.806). When gwBZ or gwBTV was combined with K-MMSE or CDR, the AUC value increased dramatically with very high sensitivity and specificity as 0.972 for gwBZ combined with K-MMSE, 0.972 for gwBTV combined with K-MMSE, 0.989 for gwBZ combined with CDR, and 0.988 for gwBTV combined with CDR.

**Table 4:** Results of receiver operating characteristic (ROC) curve analysis of gray-white matter boundary Z-score (gwBZ) and gray-white matter boundary tissue volume (gwBTV) for group classification in the hippocampus.

Indice	CN vs. MCI				CN vs. AD				MCI vs. AD			
	SE	SP	AUC	*p*	SE	SP	AUC	*p*	SE	SP	AUC	*p*
gwBZ	56.94	70.97	0.630	0.007	66.67	83.87	0.798	<0.001	50.54	83.33	0.703	<0.001
gwBTV	62.50	62.90	0.624	0.010	76.34	74.19	0.795	<0.001	43.01	90.28	0.704	<0.001
gwBZ & gwBTV	41.67	88.71	0.645	0.002	67.74	87.10	0.806	<0.001	51.61	81.94	0.703	<0.001
gwBZ & K-MMSE	56.94	85.48	0.711	<0.001	96.77	93.55	0.972	<0.001	90.32	87.50	0.925	<0.001
gwBTV & K-MMSE	59.72	80.65	0.708	<0.001	96.77	93.55	0.972	<0.001	90.32	87.50	0.924	<0.001
gwBZ & CDR	97.22	82.26	0.938	<0.001	91.40	98.39	0.989	<0.001	84.95	98.61	0.922	<0.001
gwBTV & CDR	97.22	82.26	0.939	<0.001	91.40	98.39	0.988	<0.001	84.95	98.61	0.923	<0.001
gwBZ & gwBTV & K-MMSE	55.56	85.48	0.728	<0.001	96.77	95.16	0.973	<0.001	90.32	87.50	0.926	<0.001
gwBZ & gwBTV & CDR	100.00	80.65	0.938	<0.001	92.47	96.77	0.989	<0.001	82.80	100.00	0.922	<0.001
gwBZ & gwBTV & CDR & K-MMSE	100.00	79.03	0.944	<0.001	97.85	98.39	0.998	<0.001	93.55	95.83	0.981	<0.001

We performed receiver operating characteristic (ROC) analyses using a single index of gwBZ or gwBTV value or combining gwBZ or gwBTV value with K-MMSE score or CDR score.

AUC, Area under the ROC curve; ROC, receiver operating characteristic; SE, sensitivity; SP, specificity; gwBZ, gray-white matter boundary Z-score; gwBTV, gray-white matter boundary tissue volume; K-MMSE, Korean version of the Mini-Mental State Examination; CDR, Clinical Dementia Rate; AD, Alzheimer’s disease; MCI, Mild cognitive impairment; CN, cognitively normal.

For classifying AD from MCI, AUC values of gwBZ and gwBTV were slightly lower than those for classifying AD from CN. Moreover, for classifying MCI from CN, AUC values of gwBZ and gwBTV were even lower than those for other group classifications. To enhance the presentation of the results in Table 4, we additionally added Supplementary Figure S4, which presents the graphical results of ROC analysis of gwBZ and gwBTV in the hippocampus. The true positive rate and false positive rate can be found in Table 4 as sensitivity and 100-specificity.

Supplementary Table S2 shows ROC curve analysis results in other ROIs. In the temporal lobe ROI for classifying between groups, AUC values of gwBZ and gwBTV were higher than those of hippocampus.

## Discussion

In this study, to determine if gwBZ or gwBTV could serve as an imaging biomarker for the diagnosis of AD, gwBZ and gwBTV were derived from 3D T1WI in participants of CN, MCI, and AD groups. They were then compared among the three participant groups. Their correlations with MMSE scores were then evaluated. We found two main results: First, gwBZ and gwBTV decreased in AD participants throughout the brain, showing excellent discrimination from AD to others when combined with the MMSE score. Second, gwBZ and gwBTV values were positively associated with cognitive impairment.

AD patients had significant decreases of gwBZ and gwBTV: We found that gwBZ and gwBTV values were significantly lower in AD than in MCI and CN in most of the defined ROIs (Table 2) and large brain areas in voxel-based comparison (Figure 4), suggesting that gwBZ and gwBTV decreased gradually as AD progressed. Our study was consistent with a previous study using 3D T1W images showing that gray and white matter intensity decreased near the gray-white interface in individuals with AD (Salat et al., 2011). Magnetic resonance signals of gray and white matter might be altered because of aging and diseases, thereby blurring gwB which can be mapped using the intensity of a 3D T1-weighted imaging (3D T1WI) with segmented information of both GM and WM tissues (Huppertz et al., 2008). This method is currently applied to epilepsy patients (Huppertz et al., 2008; Qu et al., 2017). Moreover, our results also showed significant differences in gwBZ and gwBTV between MCI and CN, similar to a previous study showing that gray-white matter intensity contrast ratio was lower in those with MCI who later converted to dementia than those without MCI (Jefferson et al., 2015). Alterations of gwBZ or gwBTV might precede the appearance of gray matter atrophy and might be suitable for diagnosing MCI and AD. Furthermore, gwBZ and gwBTV showed good performance for classifying AD from MCI or CN in the hippocampus (Table 4) and in the temporal lobe (Supplementary Table S2). Previous studies have found that such structural alterations directly or indirectly affect white matter integrity and gray matter metabolism changes (Villain et al., 2008) and that volumetric losses are correlated with severe neurofibrillary changes and neuronal loss (Bobinski et al., 1996). Another previous study found that microstructural changes in AD patients occur within superficial white matter along gray-white matter boundary using diffusion tensor imaging (DTI) that pointed to impaired myelination (Veale et al., 2021). N-acetyl aspartate (NAA) was also found lower in subcortical white matter using proton magnetic resonance spectroscopy (^1^H-MRS), which could negatively affect myelin repair and remyelination processes (Wong et al., 2020). Thus, changes in gwBZ and gwBTV might reflect microscopic pathology. In early stages of AD, the main manifestation is progressive atrophy of hippocampal structures caused by cell damage, axonal degeneration, and synaptic disorder. During the development of AD, brain damage mainly starts in the temporal lobe and quickly spreads along the temporal–parietal–frontal trajectory to the rest of the cortex. Brain injury caused by AD has macroscopic structural effects of cortical atrophy or degeneration of white matter tissue. Therefore, gwBZ and gwBTV that jointly exhibit gray and white matter characteristics in this study have potential as markers for diagnosing early stages of AD.

gwBZ and gwBTV had significant positive correlations with the K-MMSE score: The development of cognitive impairment was associated with decreases of gwBZ and gwBTV (Table 3, Figure 5), consistent with a previous study showing the relationship between memory impairment and the degree of gray-white matter blurring (Blackmon et al., 2019). A previous study has indicated that amyloid aggregation during cognitive impairment in AD starts in the isocortical association area, extending widely in the gray matter and penetrating into the white matter during some periods (Braak et al., 2006). Therefore, neurofibrillary tangles, which are associated with an increased risk of cognitive decline, can accumulate massively at gwB, leading to a dramatic decrease in cognitive ability in the late stage of AD. Most previous studies have focused on evaluating the correlation between the hippocampus volume and the severity of cognitive impairment and episodic memory deficits in both MCI and AD (Gordon et al., 2016). Thus, gwBZ and gwBTV might have potential to discriminate AD and other non-AD cognitive disorders.

gwBZ and gwBTV had significant negative correlations with age: Both gwBZ and gwBTV decreased with increasing age in all defined ROIs (Table 3) and widespread brain areas (Figure 5). Such negative correlation with age was also seen in MCI and AD groups, but not in the CN group (Figure 5). Previous studies have found age-related decline in T1 signal intensity in white matter and gray matter with an overall decrease in gray/white matter contrast across the cortex (Salat et al., 2009; Vidal-Pineiro et al., 2016), which might be related to diffuse myelin destruction and iron deposition over the lifespan (Bartzokis, 2004; Li et al., 2014).

Limitations of this study: The present study has several limitations. First, the spatial resolution of T1-weighted images might affect gray and white matter intensity values. Therefore, partial volume effects might slightly influence gwBZ and gwBTV values. However, this factor affects all three groups equally. Thus, significant differences between groups cannot be explained by this limitation. Second, our study did not include years of education as one of the factors of interests in AD. Although this factor modulates susceptibility to AD (Evans et al., 1997), years of education may not necessarily affect gray and white matter damage. Third, sex ratio difference might impact TIV. There were more females than males in this study. Female brain volumes are generally smaller than male brain volumes. Although we used TIV as a covariate in this experiment, it would be better to match TIV of samples in future studies. Finally, we mainly used the K-MMSE-score as an evaluation of cognitive impairment in this study. Therefore, it is necessary to investigate associations between gwBZ or gwBTV values and other cognitive task values in the future.

## Conclusion

This study found that the AD group had lower gwBZ and gwBTV than MCI and CN groups. These measures were associated with cognitive function and age in multiple brain regions. Both gwBZ and gwBTV had strong ability in discriminating AD from others, suggesting that gwBZ and gwBTV can be potential imaging biomarkers for monitoring AD progression and diagnosis. Therefore, future studies can compare characteristics of gwBZ and gwBTV to those of amyloid PET or tau PET images in AD and MCI patients.

## Data availability statement

The raw data supporting the conclusions of this article will be made available by the authors, without undue reservation.

## Ethics statement

The studies involving humans were approved by KHNMC IRB 2007-005, KHNMC IRB 2009-056, KHNMC IRB 2011-059, KHNMC IRB 2015-02-006, and KHNMC IRB 2019-07-007. The studies were conducted in accordance with the local legislation and institutional requirements. The participants provided their written informed consent to participate in this study.

## Author contributions

YT: Writing – original draft, Data curation, Formal analysis, Methodology, Software, Validation, Visualization, Writing – review & editing. J-HO: Data curation, Methodology, Software, Validation, Visualization, Writing – original draft, Writing – review & editing. HR: Data curation, Investigation, Project administration, Resources, Supervision, Validation, Writing – original draft, Writing – review & editing. SP: Data curation, Formal analysis, Investigation, Resources, Writing – review & editing. C-WR: Data curation, Formal analysis, Investigation, Resources, Writing – review & editing. AC: Data curation, Formal analysis, Investigation, Resources, Writing – review & editing. G-HJ: Conceptualization, Data curation, Formal analysis, Funding acquisition, Investigation, Methodology, Project administration, Resources, Software, Supervision, Validation, Visualization, Writing – original draft, Writing – review & editing.
