# Supplementary material for: Gray-white matter boundary Z-score and volume as imaging biomarkers of Alzheimer’s disease
Source: Front Aging Neurosci. 2023 Dec 14;15:1291376. doi: 10.3389/fnagi.2023.1291376 (PMC10755914; doi:10.3389/fnagi.2023.1291376)
Supplement: Supplementary file 1 [file Data_Sheet_1.docx]

**Gray-White Matter Boundary Z-Score and Volume as Imaging Biomarkers of Alzheimer’s Disease**

**Supplementary Tables**

**Supplementary Table S1. Results of correlation analyses of the gray-white matter boundary Z-score (gwBZ) and the gray-white boundary tissue volume (gwBTV) values with age for each group in specific areas**

| ROI | CN (r/p) | MCI(r/p) | AD(r/p) |
| --- | --- | --- | --- |
| ***gwBZ*** | | | |
| Hippocampus | r = -0.168/ p = 0.192 | r = -0.577/ p < 0.001 | r = -0.418/ p < 0.001 |
| Neocortex | r = -0.310/ p = 0.014 | r = -0.552/ p < 0.001 | r = -0.328/ p = 0.001 |
| Cuneus | r = -0.351/ p = 0.005 | r = -0.662/ p < 0.001 | r = -0.398/ p < 0.001 |
| Insula | r = -0.254/ p = 0.046 | r = -0.507/ p < 0.001 | r = -0.413/ p < 0.001 |
| MTG | r = -0.083/ p = 0.521 | r = -0.243/ p = 0.040 | r = -0.012/ p = 0.912 |
| Parahippocampal | r = -0.111/ p = 0.391 | r = -0.512/ p < 0.001 | r = -0.350/ p < 0.001 |
| Precuneus | r = -0.280/ p = 0.028 | r = -0.517/ p < 0.001 | r = -0.201/ p = 0.053 |
| Thalamus | r = -0.334/ p = 0.008 | r = -0.544/ p < 0.001 | r = -0.392/ p < 0.001 |
| Frontal lobe | r = -0.348/ p = 0.006 | r = -0.457/ p < 0.001 | r = -0.200/ p = 0.054 |
| Limbic lobe | r = -0.202/ p = 0.115 | r = -0.514/ p < 0.001 | r = -0.323/ p = 0.002 |
| Occipital lobe | r = -0.305/ p = 0.016 | r = -0.636/ p < 0.001 | r = -0.334/ p = 0.001 |
| Parietal lobe | r = -0.297/ p = 0.019 | r = -0.490/ p < 0.001 | r = -0.157/ p = 0.134 |
| Temporal lobe | r = -0.174/ p = 0.176 | r = -0.501/ p < 0.001 | r = -0.142/ p = 0.176 |
| ***gwBTV*** | | | |
| Hippocampus | r = -0.175/ p = 0.174 | r = -0.578/ p < 0.001 | r = -0.418/ p < 0.001 |
| Neocortex | r = -0.296/ p = 0.020 | r = -0.579/ p < 0.001 | r = -0.322/ p = 0.002 |
| Cuneus | r = -0.352/ p = 0.005 | r = -0.675/ p < 0.001 | r = -0.405/ p < 0.001 |
| Insula | r = -0.262/ p = 0.040 | r = -0.515/ p < 0.001 | r = -0.422/ p < 0.001 |
| MTG | r = -0.044/ p = 0.734 | r = -0.215/ p = 0.070 | r = -0.010/ p = 0.926 |
| Parahippocampal | r = -0.102/ p = 0.430 | r = -0.509/ p < 0.001 | r = -0.326/ p = 0.001 |
| Precuneus | r = -0.282/ p = 0.026 | r = -0.521/ p < 0.001 | r = -0.202/ p = 0.051 |
| Thalamus | r = -0.336/ p = 0.008 | r = -0.541/ p < 0.001 | r = -0.394/ p < 0.001 |
| Frontal lobe | r = -0.374/ p = 0.003 | r = -0.487/ p < 0.001 | r = -0.213/ p = 0.040 |
| Limbic lobe | r = -0.181/ p = 0.159 | r = -0.518/ p < 0.001 | r = -0.299/ p = 0.004 |
| Occipital lobe | r = -0.300/ p = 0.019 | r = -0.615/ p < 0.001 | r = -0.334/ p = 0.001 |
| Parietal lobe | r = -0.305/ p = 0.016 | r = -0.492/ p < 0.001 | r = -0.176/ p = 0.092 |
| Temporal lobe | r = -0.139/ p = 0.280 | r = -0.494/ p < 0.001 | r = -0.130/ p = 0.214 |

The data of age are listed as correlation coefficient (r) and p-value by correlation analysis.

gwB, gray-white matter boundary; gwBZ, gray-white matter boundary Z-score; gwBTV, gray-white matter boundary tissue volume; ROI, regions-of-interest; CN, cognitively normal; MCI, mild cognitive impairment; AD, Alzheimer’s disease; MTG, middle temporal gyrus.

**Supplementary Table S2. Results of the receiver operating characteristic (ROC) curve analysis of the gray-white matter boundary Z-score (gwBZ) and gray-white matter boundary tissue volume (gwBTV) to evaluate the group classification in specific areas.**

| ROI | | CN vs MCI | | | | CN vs AD | | | | MCI vs AD | | | |
| --- | --- | --- | --- | --- | --- | --- | --- | --- | --- | --- | --- | --- | --- |
|  |  | SE | SP | AUC | p | SE | SP | AUC | p | SE | SP | AUC | p |
| Neocortex | gwBZ | 79.17 | 45.16 | 0.621 | 0.013 | 95.70 | 45.16 | 0.769 | <0.001 | 46.24 | 83.33 | 0.678 | <0.001 |
|  | gwBTV | 73.61 | 48.39 | 0.622 | 0.012 | 70.97 | 70.97 | 0.782 | <0.001 | 47.31 | 81.94 | 0.691 | <0.001 |
|  | gwBZ&gwBTV | 84.72 | 43.55 | 0.621 | 0.014 | 87.10 | 61.29 | 0.807 | <0.001 | 48.39 | 84.72 | 0.725 | <0.001 |
|  | gwBZ & K-MMSE | 63.89 | 75.81 | 0.714 | <0.001 | 93.55 | 95.16 | 0.973 | <0.001 | 90.32 | 86.11 | 0.921 | <0.001 |
|  | gwBTV & K-MMSE | 66.67 | 70.97 | 0.710 | <0.001 | 93.55 | 95.16 | 0.973 | <0.001 | 91.40 | 86.11 | 0.923 | <0.001 |
|  | gwBZ & CDR | 94.44 | 87.10 | 0.951 | <0.001 | 97.85 | 95.16 | 0.993 | <0.001 | 84.95 | 100.00 | 0.920 | <0.001 |
|  | gwBTV & CDR | 95.83 | 87.10 | 0.950 | <0.001 | 97.85 | 95.16 | 0.993 | <0.001 | 84.95 | 100.00 | 0.920 | <0.001 |
|  | gwBZ & gwBTV & K-MMSE | 68.06 | 72.58 | 0.715 | <0.001 | 93.55 | 95.16 | 0.973 | <0.001 | 88.17 | 87.50 | 0.924 | <0.001 |
|  | gwBZ & gwBTV & CDR | 94.44 | 87.10 | 0.952 | <0.001 | 97.85 | 95.16 | 0.993 | <0.001 | 84.95 | 100.00 | 0.922 | <0.001 |
|  | gwBZ & gwBTV & CDR & K-MMSE | 91.67 | 93.55 | 0.956 | <0.001 | 98.92 | 98.39 | 0.998 | <0.001 | 93.55 | 95.83 | 0.982 | <0.001 |
| Cuneus | gwBZ | 86.11 | 27.42 | 0.543 | 0.393 | 63.44 | 64.52 | 0.663 | <0.001 | 37.63 | 84.72 | 0.625 | 0.004 |
|  | gwBTV | 58.33 | 54.84 | 0.544 | 0.378 | 74.19 | 54.84 | 0.668 | <0.001 | 46.24 | 76.39 | 0.632 | 0.002 |
|  | gwBZ&gwBTV | 55.56 | 58.06 | 0.550 | 0.322 | 78.49 | 50.00 | 0.672 | <0.001 | 79.57 | 45.83 | 0.646 | <0.001 |
|  | gwBZ & K-MMSE | 75.00 | 53.23 | 0.678 | <0.001 | 95.70 | 88.71 | 0.963 | <0.001 | 83.87 | 86.11 | 0.909 | <0.001 |
|  | gwBTV & K-MMSE | 75.00 | 53.23 | 0.677 | <0.001 | 95.70 | 88.71 | 0.963 | <0.001 | 83.87 | 86.11 | 0.908 | <0.001 |
|  | gwBZ & CDR | 100.00 | 79.03 | 0.924 | <0.001 | 94.62 | 93.55 | 0.989 | <0.001 | 82.80 | 100.00 | 0.922 | <0.001 |
|  | gwBTV & CDR | 100.00 | 79.03 | 0.922 | <0.001 | 94.62 | 93.55 | 0.988 | <0.001 | 82.80 | 100.00 | 0.924 | <0.001 |
|  | gwBZ & gwBTV & K-MMSE | 41.67 | 87.10 | 0.685 | <0.001 | 94.62 | 91.94 | 0.964 | <0.001 | 92.47 | 77.78 | 0.909 | <0.001 |
|  | gwBZ & gwBTV & CDR | 100.00 | 79.03 | 0.923 | <0.001 | 96.77 | 93.55 | 0.989 | <0.001 | 82.80 | 100.00 | 0.932 | <0.001 |
|  | gwBZ & gwBTV & CDR & K-MMSE | 97.22 | 82.26 | 0.931 | <0.001 | 98.92 | 98.39 | 0.998 | <0.001 | 96.77 | 91.67 | 0.984 | <0.001 |
| Insula | gwBZ | 70.83 | 56.45 | 0.614 | 0.022 | 86.02 | 61.29 | 0.779 | <0.001 | 65.59 | 68.06 | 0.715 | <0.001 |
|  | gwBTV | 66.67 | 58.06 | 0.605 | 0.036 | 88.17 | 58.06 | 0.771 | <0.001 | 70.97 | 63.89 | 0.709 | <0.001 |
|  | gwBZ&gwBTV | 54.17 | 72.58 | 0.656 | 0.001 | 63.44 | 87.10 | 0.804 | <0.001 | 66.67 | 69.44 | 0.718 | <0.001 |
|  | gwBZ & K-MMSE | 66.67 | 77.42 | 0.711 | <0.001 | 95.70 | 91.94 | 0.973 | <0.001 | 92.47 | 86.11 | 0.929 | <0.001 |
|  | gwBTV & K-MMSE | 65.28 | 74.19 | 0.707 | <0.001 | 93.55 | 95.16 | 0.973 | <0.001 | 92.47 | 86.11 | 0.929 | <0.001 |
|  | gwBZ & CDR | 94.44 | 88.71 | 0.948 | <0.001 | 95.70 | 96.77 | 0.993 | <0.001 | 86.02 | 100.00 | 0.931 | <0.001 |
|  | gwBTV & CDR | 94.44 | 88.71 | 0.945 | <0.001 | 95.70 | 96.77 | 0.993 | <0.001 | 86.02 | 100.00 | 0.931 | <0.001 |
|  | gwBZ & gwBTV & K-MMSE | 52.78 | 85.48 | 0.716 | <0.001 | 95.70 | 91.94 | 0.974 | <0.001 | 92.47 | 86.11 | 0.929 | <0.001 |
|  | gwBZ & gwBTV & CDR | 95.83 | 85.48 | 0.953 | <0.001 | 94.62 | 98.39 | 0.993 | <0.01 | 86.02 | 100.00 | 0.932 | <0.001 |
|  | gwBZ & gwBTV & CDR & K-MMSE | 97.22 | 87.10 | 0.955 | <0.001 | 98.92 | 98.39 | 0.999 | <0.001 | 96.77 | 94.44 | 0.984 | <0.001 |
| MTG | gwBZ | 52.78 | 74.19 | 0.668 | <0.001 | 82.80 | 53.23 | 0.742 | <0.001 | 43.01 | 73.61 | 0.590 | 0.043 |
|  | gwBTV | 69.44 | 59.68 | 0.678 | <0.001 | 49.46 | 87.10 | 0.737 | <0.001 | 45.16 | 72.22 | 0.580 | 0.073 |
|  | gwBZ&gwBTV | 69.44 | 64.52 | 0.696 | <0.001 | 48.39 | 87.10 | 0.736 | <0.001 | 87.10 | 33.33 | 0.594 | 0.037 |
|  | gwBZ & K-MMSE | 65.28 | 74.19 | 0.747 | <0.001 | 92.47 | 96.77 | 0.967 | <0.001 | 88.17 | 84.72 | 0.909 | <0.001 |
|  | gwBTV & K-MMSE | 66.67 | 75.81 | 0.756 | <0.001 | 93.55 | 95.16 | 0.968 | <0.001 | 88.17 | 84.72 | 0.910 | <0.001 |
|  | gwBZ & CDR | 93.06 | 88.71 | 0.946 | <0.001 | 92.47 | 98.39 | 0.992 | <0.001 | 82.80 | 100.00 | 0.923 | <0.001 |
|  | gwBTV & CDR | 94.44 | 87.10 | 0.949 | <0.001 | 92.47 | 98.39 | 0.992 | <0.001 | 82.80 | 100.00 | 0.922 | <0.001 |
|  | gwBZ & gwBTV & K-MMSE | 84.72 | 59.68 | 0.771 | <0.001 | 92.47 | 95.16 | 0.968 | <0.001 | 88.17 | 84.72 | 0.910 | <0.001 |
|  | gwBZ & gwBTV & CDR | 98.16 | 82.26 | 0.951 | <0.001 | 92.47 | 98.39 | 0.993 | <0.001 | 82.80 | 100.00 | 0.930 | <0.001 |
|  | gwBZ & gwBTV & CDR & K-MMSE | 95.83 | 87.10 | 0.959 | <0.001 | 98.92 | 98.39 | 0.998 | <0.001 | 89.25 | 98.61 | 0.984 | <0.001 |
| Parahippocampal | gwBZ | 47.22 | 74.19 | 0.620 | 0.013 | 43.01 | 95.16 | 0.747 | <0.001 | 37.63 | 88.89 | 0.641 | 0.001 |
|  | gwBTV | 27.78 | 93.55 | 0.623 | 0.011 | 46.24 | 93.55 | 0.750 | <0.001 | 39.78 | 88.89 | 0.638 | 0.001 |
|  | gwBZ&gwBTV | 75.00 | 51.61 | 0.623 | 0.012 | 44.09 | 95.16 | 0.756 | <0.001 | 40.86 | 86.11 | 0.643 | <0.001 |
|  | gwBZ & K-MMSE | 58.33 | 83.87 | 0.707 | <0.001 | 97.85 | 90.32 | 0.975 | <0.001 | 90.32 | 86.11 | 0.923 | <0.001 |
|  | gwBTV & K-MMSE | 58.33 | 83.87 | 0.707 | <0.001 | 92.47 | 95.16 | 0.976 | <0.001 | 88.17 | 88.89 | 0.922 | <0.001 |
|  | gwBZ & CDR | 98.61 | 80.65 | 0.942 | <0.001 | 96.77 | 93.55 | 0.991 | <0.001 | 82.80 | 100.00 | 0.914 | <0.001 |
|  | gwBTV & CDR | 98.61 | 80.65 | 0.943 | <0.001 | 96.77 | 93.55 | 0.991 | <0.001 | 82.80 | 100.00 | 0.912 | <0.001 |
|  | gwBZ & gwBTV & K-MMSE | 69.44 | 70.97 | 0.708 | <0.001 | 92.47 | 95.16 | 0.976 | <0.001 | 90.32 | 87.50 | 0.923 | <0.001 |
|  | gwBZ & gwBTV & CDR | 100.00 | 79.03 | 0.947 | <0.001 | 97.85 | 91.94 | 0.991 | <0.001 | 84.95 | 100.00 | 0.925 | <0.001 |
|  | gwBZ & gwBTV & CDR & K-MMSE | 95.83 | 83.87 | 0.954 | <0.001 | 96.77 | 100.00 | 0.999 | <0.001 | 97.85 | 91.67 | 0.983 | <0.001 |
| Precuneus | gwBZ | 37.50 | 83.87 | 0.598 | 0.047 | 53.76 | 75.81 | 0.674 | <0.001 | 39.78 | 76.39 | 0.582 | 0.069 |
|  | gwBTV | 38.89 | 83.87 | 0.602 | 0.037 | 46.24 | 83.87 | 0.677 | <0.001 | 37.63 | 79.17 | 0.584 | 0.060 |
|  | gwBZ&gwBTV | 66.67 | 59.68 | 0.633 | 0.006 | 64.52 | 70.97 | 0.691 | <0.001 | 37.63 | 80.56 | 0.583 | 0.062 |
|  | gwBZ & K-MMSE | 56.94 | 79.03 | 0.688 | <0.001 | 91.40 | 93.55 | 0.963 | <0.001 | 86.02 | 86.11 | 0.910 | <0.001 |
|  | gwBTV & K-MMSE | 55.56 | 80.65 | 0.690 | <0.001 | 93.55 | 91.94 | 0.963 | <0.001 | 86.02 | 86.11 | 0.910 | <0.001 |
|  | gwBZ & CDR | 95.83 | 85.45 | 0.934 | <0.001 | 96.77 | 96.77 | 0.994 | <0.001 | 82.80 | 100.00 | 0.942 | <0.001 |
|  | gwBTV & CDR | 100.00 | 82.26 | 0.933 | <0.001 | 98.92 | 93.55 | 0.994 | <0.001 | 82.80 | 100.00 | 0.943 | <0.001 |
|  | gwBZ & gwBTV & K-MMSE | 63.89 | 70.97 | 0.708 | <0.001 | 91.40 | 93.55 | 0.963 | <0.001 | 89.25 | 81.94 | 0.909 | <0.001 |
|  | gwBZ & gwBTV & CDR | 97.22 | 85.48 | 0.933 | <0.001 | 96.77 | 96.77 | 0.995 | <0.001 | 82.80 | 100.00 | 0.942 | <0.001 |
|  | gwBZ & gwBTV & CDR & K-MMSE | 100.00 | 80.65 | 0.942 | <0.001 | 98.92 | 100.00 | 0.999 | <0.001 | 92.47 | 97.22 | 0.988 | <0.001 |
| Thalamus | gwBZ | 40.28 | 88.71 | 0.634 | 0.005 | 60.22 | 88.71 | 0.814 | <0.001 | 83.87 | 50.00 | 0.699 | <0.001 |
|  | gwBTV | 37.50 | 88.71 | 0.627 | 0.008 | 60.22 | 87.10 | 0.809 | <0.001 | 78.49 | 54.17 | 0.697 | <0.001 |
|  | gwBZ&gwBTV | 66.67 | 62.90 | 0.663 | <0.001 | 80.65 | 79.03 | 0.849 | <0.001 | 65.59 | 68.06 | 0.705 | <0.001 |
|  | gwBZ & K-MMSE | 55.56 | 83.87 | 0.710 | <0.001 | 94.62 | 91.94 | 0.974 | <0.001 | 91.40 | 84.72 | 0.920 | <0.001 |
|  | gwBTV & K-MMSE | 58.33 | 79.03 | 0.706 | <0.001 | 97.85 | 88.71 | 0.974 | <0.001 | 89.25 | 86.11 | 0.919 | <0.001 |
|  | gwBZ & CDR | 100.00 | 80.65 | 0.938 | <0.001 | 100.00 | 100.00 | 1.000 | <0.001 | 82.80 | 100.00 | 0.924 | <0.001 |
|  | gwBTV & CDR | 100.00 | 80.65 | 0.937 | <0.001 | 93.55 | 96.77 | 0.990 | <0.001 | 82.80 | 100.00 | 0.922 | <0.001 |
|  | gwBZ & gwBTV & K-MMSE | 56.94 | 83.87 | 0.724 | <0.001 | 92.47 | 95.16 | 0.979 | <0.001 | 90.32 | 84.72 | 0.922 | <0.001 |
|  | gwBZ & gwBTV & CDR | 100.00 | 80.65 | 0.938 | <0.001 | 93.55 | 98.39 | 0.991 | <0.001 | 82.80 | 100.00 | 0.938 | <0.001 |
|  | gwBZ & gwBTV & CDR & K-MMSE | 98.61 | 80.65 | 0.945 | <0.001 | 97.85 | 100.00 | 0.999 | <0.001 | 94.62 | 94.44 | 0.983 | <0.001 |
| Frontal lobe | gwBZ | 69.44 | 54.84 | 0.597 | 0.051 | 78.49 | 53.23 | 0.659 | <0.001 | 58.06 | 56.94 | 0.568 | 0.129 |
|  | gwBTV | 68.06 | 54.84 | 0.607 | 0.030 | 75.27 | 54.84 | 0.662 | <0.001 | 29.17 | 83.87 | 0.565 | 0.146 |
|  | gwBZ&gwBTV | 61.11 | 66.13 | 0.622 | 0.013 | 69.89 | 58.06 | 0.667 | <0.001 | 60.22 | 54.17 | 0.570 | 0.121 |
|  | gwBZ & K-MMSE | 61.11 | 75.81 | 0.698 | <0.001 | 94.62 | 91.94 | 0.974 | <0.001 | 91.40 | 93.55 | 0.964 | <0.001 |
|  | gwBTV & K-MMSE | 59.72 | 75.81 | 0.701 | <0.001 | 91.40 | 93.55 | 0.965 | <0.001 | 90.32 | 80.56 | 0.909 | <0.001 |
|  | gwBZ & CDR | 98.61 | 83.87 | 0.945 | <0.001 | 97.85 | 93.55 | 0.991 | <0.001 | 82.80 | 100.00 | 0.919 | <0.001 |
|  | gwBTV & CDR | 100.00 | 83.87 | 0.949 | <0.001 | 97.85 | 93.55 | 0.992 | <0.001 | 83.87 | 100.00 | 0.918 | <0.001 |
|  | gwBZ & gwBTV & K-MMSE | 58.33 | 80.65 | 0.715 | <0.001 | 91.40 | 93.55 | 0.965 | <0.001 | 90.32 | 80.56 | 0.907 | <0.001 |
|  | gwBZ & gwBTV & CDR | 98.61 | 88.71 | 0.953 | <0.001 | 97.85 | 93.55 | 0.993 | <0.001 | 82.80 | 100.00 | 0.920 | <0.001 |
|  | gwBZ & gwBTV & CDR & K-MMSE | 100.00 | 90.32 | 0.958 | <0.001 | 98.92 | 98.39 | 0.998 | <0.001 | 91.40 | 98.61 | 0.982 | <0.001 |
| Limbic lobe | gwBZ | 48.61 | 79.03 | 0.647 | 0.002 | 59.14 | 79.03 | 0.749 | <0.001 | 30.11 | 95.83 | 0.608 | 0.014 |
|  | gwBTV | 77.78 | 50.00 | 0.651 | 0.002 | 92.47 | 46.77 | 0.749 | <0.001 | 29.03 | 97.22 | 0.612 | 0.010 |
|  | gwBZ&gwBTV | 61.11 | 69.35 | 0.652 | 0.001 | 94.62 | 48.39 | 0.753 | <0.001 | 34.41 | 95.83 | 0.630 | 0.003 |
|  | gwBZ & K-MMSE | 62.50 | 82.26 | 0.724 | <0.001 | 93.55 | 95.16 | 0.974 | <0.001 | 93.55 | 95.16 | 0.974 | <0.001 |
|  | gwBTV & K-MMSE | 61.11 | 82.26 | 0.724 | <0.001 | 92.47 | 95.16 | 0.974 | <0.001 | 93.55 | 84.72 | 0.919 | <0.001 |
|  | gwBZ & CDR | 100.00 | 80.65 | 0.948 | <0.001 | 96.77 | 95.16 | 0.992 | <0.001 | 83.87 | 100.00 | 0.912 | <0.001 |
|  | gwBTV & CDR | 100.00 | 80.65 | 0.949 | <0.001 | 96.77 | 95.16 | 0.992 | <0.001 | 83.87 | 100.00 | 0.911 | <0.001 |
|  | gwBZ & gwBTV & K-MMSE | 63.89 | 80.65 | 0.727 | <0.001 | 93.55 | 93.55 | 0.974 | <0.001 | 93.55 | 84.72 | 0.920 | <0.001 |
|  | gwBZ & gwBTV & CDR | 100.00 | 80.65 | 0.948 | <0.001 | 96.77 | 95.16 | 0.992 | <0.001 | 83.87 | 100.00 | 0.917 | <0.001 |
|  | gwBZ & gwBTV & CDR & K-MMSE | 86.11 | 93.55 | 0.955 | <0.001 | 98.92 | 98.39 | 0.999 | <0.001 | 95.70 | 95.83 | 0.984 | <0.001 |
| Occipital lobe | gwBZ | 88.89 | 22.58 | 0.534 | 0.493 | 77.42 | 56.45 | 0.696 | <0.001 | 77.42 | 51.39 | 0.662 | <0.001 |
|  | gwBTV | 58.33 | 52.23 | 0.541 | 0.408 | 78.49 | 56.45 | 0.703 | <0.001 | 67.74 | 63.89 | 0.669 | <0.001 |
|  | gwBZ&gwBTV | 33.33 | 79.03 | 0.559 | 0.235 | 78.49 | 66.13 | 0.733 | <0.001 | 73.12 | 58.33 | 0.670 | <0.001 |
|  | gwBZ & K-MMSE | 56.94 | 72.58 | 0.676 | <0.001 | 91.40 | 93.55 | 0.962 | <0.001 | 91.40 | 93.55 | 0.962 | <0.001 |
|  | gwBTV & K-MMSE | 56.94 | 72.58 | 0.678 | <0.001 | 93.55 | 90.32 | 0.963 | <0.001 | 86.02 | 84.72 | 0.910 | <0.001 |
|  | gwBZ & CDR | 100.00 | 79.03 | 0.923 | <0.001 | 93.55 | 96.77 | 0.991 | <0.001 | 82.80 | 100.00 | 0.936 | <0.001 |
|  | gwBTV & CDR | 100.00 | 79.03 | 0.926 | <0.001 | 94.62 | 96.77 | 0.992 | <0.001 | 82.80 | 100.00 | 0.937 | <0.001 |
|  | gwBZ & gwBTV & K-MMSE | 63.89 | 66.13 | 0.678 | <0.001 | 93.55 | 90.32 | 0.963 | <0.001 | 86.02 | 84.72 | 0.910 | <0.001 |
|  | gwBZ & gwBTV & CDR | 100.00 | 79.03 | 0.926 | <0.001 | 94.62 | 96.77 | 0.991 | <0.001 | 82.80 | 100.00 | 0.936 | <0.001 |
|  | gwBZ & gwBTV & CDR & K-MMSE | 100.00 | 80.65 | 0.936 | <0.001 | 98.92 | 98.39 | 0.998 | <0.001 | 92.47 | 97.22 | 0.985 | <0.001 |
| Parietal lobe | gwBZ | 44.44 | 72.58 | 0.569 | 0.166 | 59.14 | 72.58 | 0.679 | <0.001 | 67.74 | 51.39 | 0.612 | 0.012 |
|  | gwBTV | 68.06 | 48.39 | 0.579 | 0.112 | 62.37 | 67.74 | 0.678 | <0.001 | 86.02 | 30.56 | 0.594 | 0.039 |
|  | gwBZ&gwBTV | 62.50 | 64.52 | 0.644 | 0.003 | 68.82 | 59.68 | 0.679 | <0.001 | 90.32 | 37.50 | 0.654 | <0.001 |
|  | gwBZ & K-MMSE | 69.44 | 64.52 | 0.685 | <0.001 | 92.47 | 93.55 | 0.964 | <0.001 | 92.47 | 93.55 | 0.964 | <0.001 |
|  | gwBTV & K-MMSE | 55.56 | 80.65 | 0.690 | <0.001 | 92.47 | 93.55 | 0.964 | <0.001 | 84.95 | 87.50 | 0.910 | <0.001 |
|  | gwBZ & CDR | 100.00 | 80.65 | 0.928 | <0.001 | 98.92 | 93.55 | 0.993 | <0.001 | 82.80 | 100.00 | 0.944 | <0.001 |
|  | gwBTV & CDR | 100.00 | 82.26 | 0.929 | <0.001 | 100.00 | 93.55 | 0.993 | <0.001 | 82.80 | 100.00 | 0.942 | <0.001 |
|  | gwBZ & gwBTV & K-MMSE | 76.39 | 59.68 | 0.721 | <0.001 | 93.55 | 91.94 | 0.964 | <0.001 | 90.32 | 80.56 | 0.915 | <0.001 |
|  | gwBZ & gwBTV & CDR | 98.61 | 85.48 | 0.936 | <0.001 | 97.85 | 93.55 | 0.993 | <0.001 | 84.95 | 98.61 | 0.952 | <0.001 |
|  | gwBZ & gwBTV & CDR & K-MMSE | 97.22 | 87.10 | 0.947 | <0.001 | 98.92 | 98.39 | 0.998 | <0.001 | 90.32 | 98.61 | 0.986 | <0.001 |
| Temporal lobe | gwBZ | 62.50 | 69.35 | 0.691 | <0.001 | 80.65 | 72.58 | 0.829 | <0.001 | 91.40 | 34.72 | 0.672 | <0.001 |
|  | gwBTV | 65.28 | 66.13 | 0.694 | <0.001 | 60.22 | 88.71 | 0.821 | <0.001 | 39.78 | 87.50 | 0.667 | <0.001 |
|  | gwBZ&gwBTV | 70.83 | 59.68 | 0.690 | <0.001 | 80.65 | 74.19 | 0.831 | <0.001 | 91.40 | 38.89 | 0.674 | <0.001 |
|  | gwBZ & K-MMSE | 68.06 | 74.19 | 0.752 | <0.001 | 92.47 | 96.77 | 0.973 | <0.001 | 92.47 | 96.77 | 0.973 | <0.001 |
|  | gwBTV & K-MMSE | 66.67 | 77.42 | 0.756 | <0.001 | 93.55 | 96.77 | 0.974 | <0.001 | 89.25 | 84.72 | 0.917 | <0.001 |
|  | gwBZ & CDR | 91.67 | 91.94 | 0.957 | <0.001 | 100.00 | 93.55 | 0.995 | <0.001 | 82.80 | 100.00 | 0.933 | <0.001 |
|  | gwBTV & CDR | 97.22 | 87.10 | 0.954 | <0.001 | 98.92 | 93.55 | 0.995 | <0.001 | 82.80 | 100.00 | 0.932 | <0.001 |
|  | gwBZ & gwBTV & K-MMSE | 58.33 | 83.87 | 0.762 | <0.001 | 94.62 | 96.77 | 0.975 | <0.001 | 86.02 | 91.67 | 0.934 | <0.001 |
|  | gwBZ & gwBTV & CDR | 97.22 | 87.10 | 0.955 | <0.001 | 98.92 | 93.55 | 0.995 | <0.001 | 92.47 | 100.00 | 0.941 | <0.001 |
|  | gwBZ & gwBTV & CDR & K-MMSE | 97.22 | 90.32 | 0.958 | <0.001 | 98.92 | 98.39 | 0.998 | <0.001 | 91.40 | 97.22 | 0.983 | <0.001 |

Receiver operating characteristic (ROC) analyses was performed with the gwBZ or gwBTV value and with combining the gwBZ or gwBTV value with K-MMSE or CDR in each regions-of-interest (ROI).

ROI, regions-of-interest; CN, cognitively normal; MCI, mild cognitive impairment; AD, Alzheimer’s disease; gwBZ, gray-white matter boundary Z-score; gwBTV, gray-white matter boundary tissue volume; MTG, middle temporal gyrus; K-MMSE, Korean version of the Mini-Mental State Examination; CDR, Clinical Dementia Rate.

**Supplementary Figures**


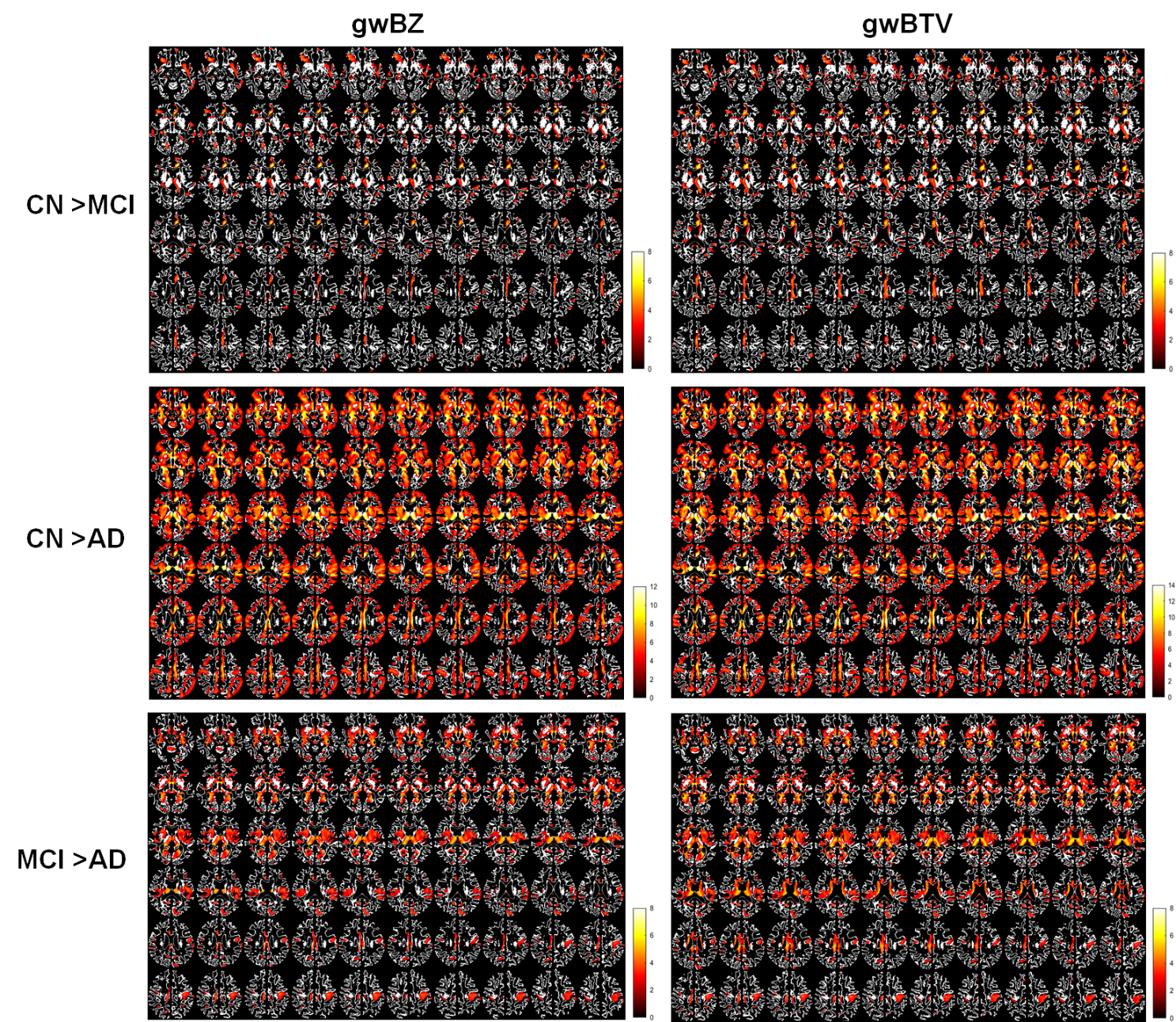


**Supplementary Figure S1**. **Overlay into the axial slices of the gray-white matter boundary map of Figure 4, which are results of the voxel-based analysis of covariance (ANCOVA) of the gray-white matter boundary Z-score (gwBZ) and gray-white matter boundary tissue volume (gwBTV) among the three participant groups.**

Red color indicates the significant difference areas in the level of gray-white matter boundary. Statistical significance was reached if p < 0.01, false discovery rate corrected. The color bar presents the T-value under significant difference areas.

CN, cognitively normal; MCI, mild cognitive impairment; AD, Alzheimer’s disease; gwBZ, gray-white matter boundary Z-score; gwBTV, gray-white matter boundary tissue volume.


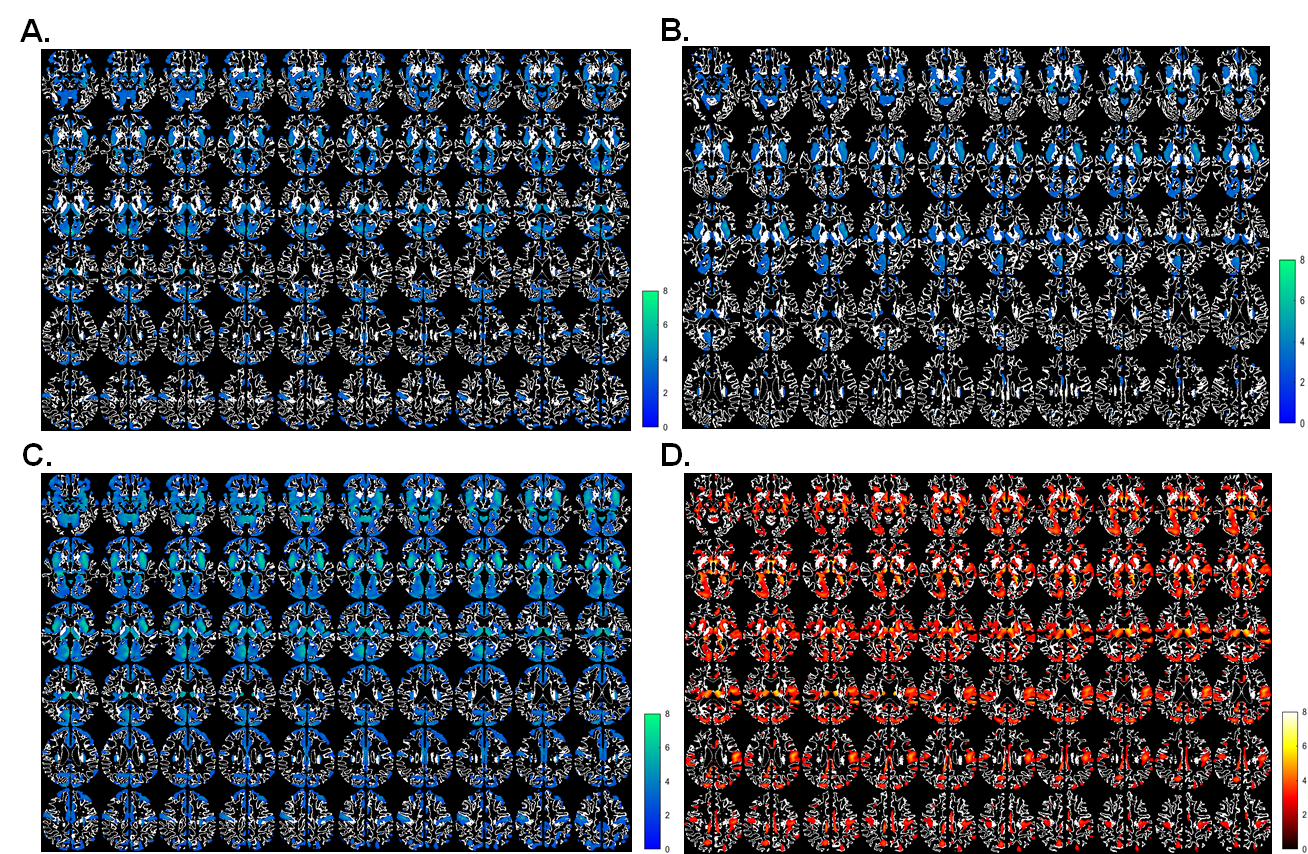


**Supplementary Figure S2**. **Overlay into the axial slices of the gray-white matter boundary map of Figure 5, which are results of voxel-based multiple regression analysis of the gray-white matter boundary Z-score (gwBZ) with age or Korean version of the Mini-Mental State Examination (K-MMSE).**

Blue color indicates areas where is a negative correlation between gwBZ with age in (A) MCI, (B) AD and (C) all groups. Red color indicates areas where is a positive correlation between gwBZ with K-MMSE in (D) all groups. Statistical significance was reached if p < 0.01, false discovery rate corrected. The color bar presents the T-value under significant difference areas.

CN, cognitively normal; MCI, mild cognitive impairment; AD, Alzheimer’s disease; gwB, gray-white matter boundary; gwBZ, gray-white matter boundary Z-score; K-MMSE, Korean version of the Mini-Mental State Examination.


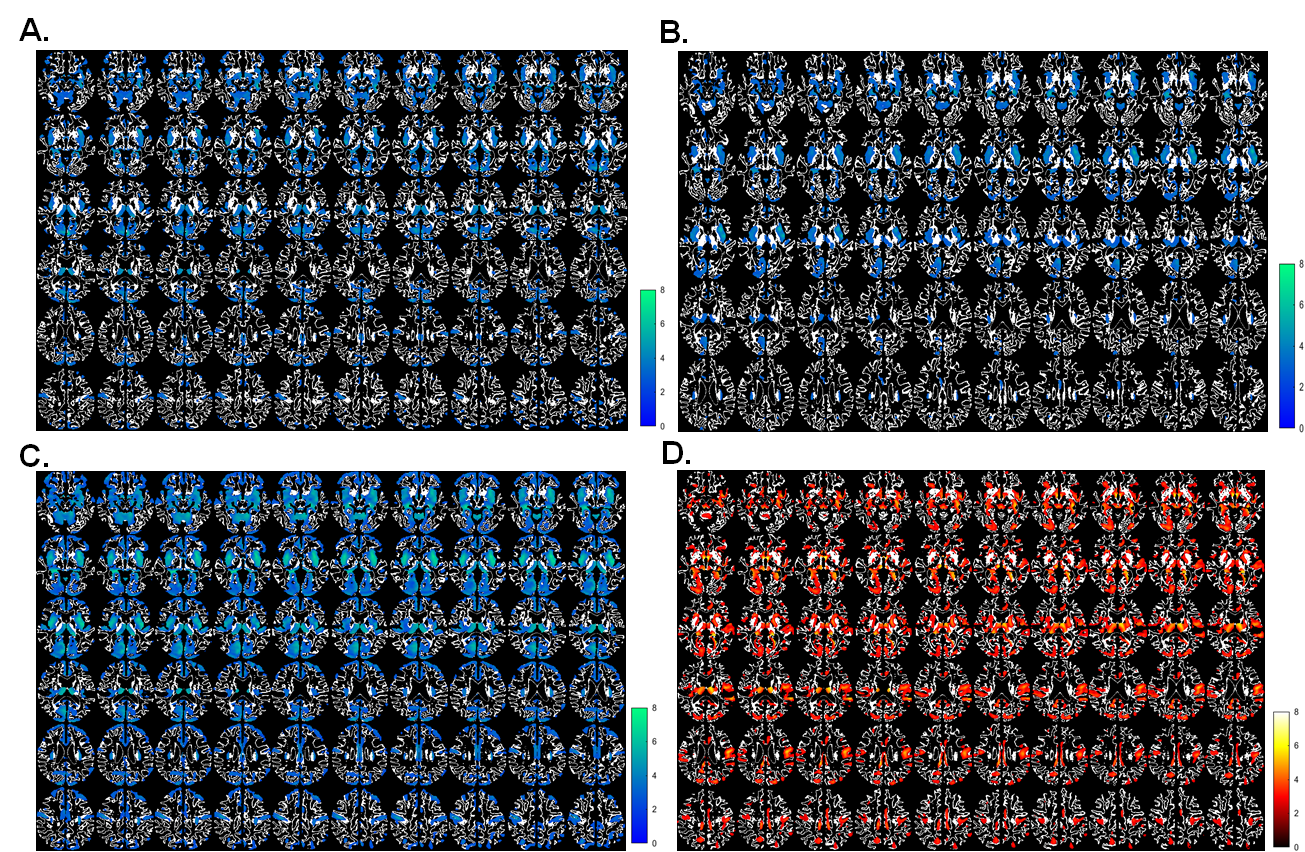


**Supplementary Figure S3**. **Overlay into the axial slices of the gray-white matter boundary map of Figure 5, which are results of voxel-based multiple regression analysis of gray-white matter boundary tissue volume (gwBTV) with age or Korean version of the Mini-Mental State Examination (K-MMSE) in the level of gray-white matter boundary (gwB).**

Blue color indicates areas where is a negative correlation between gwBTV with age in (A) MCI, (B) AD and (C) all groups. Red color indicates areas where is a positive correlation between gwBTV with K-MMSE in (D) all groups. Statistical significance was reached if p < 0.01, false discovery rate corrected. The color bar presents the T-value under significant difference areas.

CN, cognitively normal; MCI, mild cognitive impairment; AD, Alzheimer’s disease; gwB, gray-white matter boundary; gwBTV, gray-white matter boundary tissue volume; K-MMSE, Korean version of the Mini-Mental State Examination.


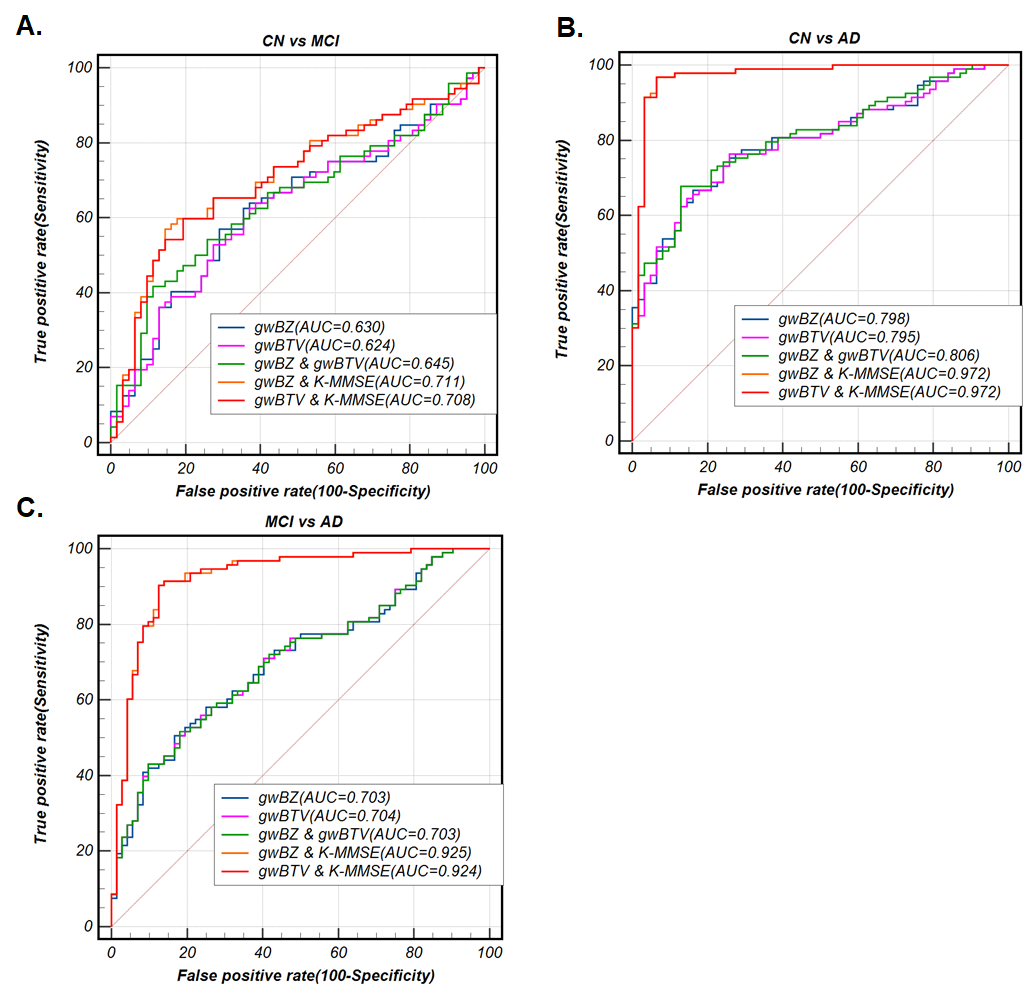


**Supplementary Figure S4. Graphical illustration of Table 4, which lists results of receiver operating characteristic (ROC) curves analysis of gray-white matter boundary Z-score (gwBZ) and its tissue volume (gwBTV) or in combination with Korean version of the Mini-Mental State Examination (K-MMSE).**

(A) Comparison of AUCs to distinguish MCI from CN, (B) Comparison of AUCs to distinguish AD from CN, and (C) Comparison of AUCs to distinguish AD from MCI.

AUC, area under the curve; AD, Alzheimer's disease; CN, cognitively normal; MCI, mild cognitive impairment; K-MMSE, Korean version of the Mini-Mental State Examination.


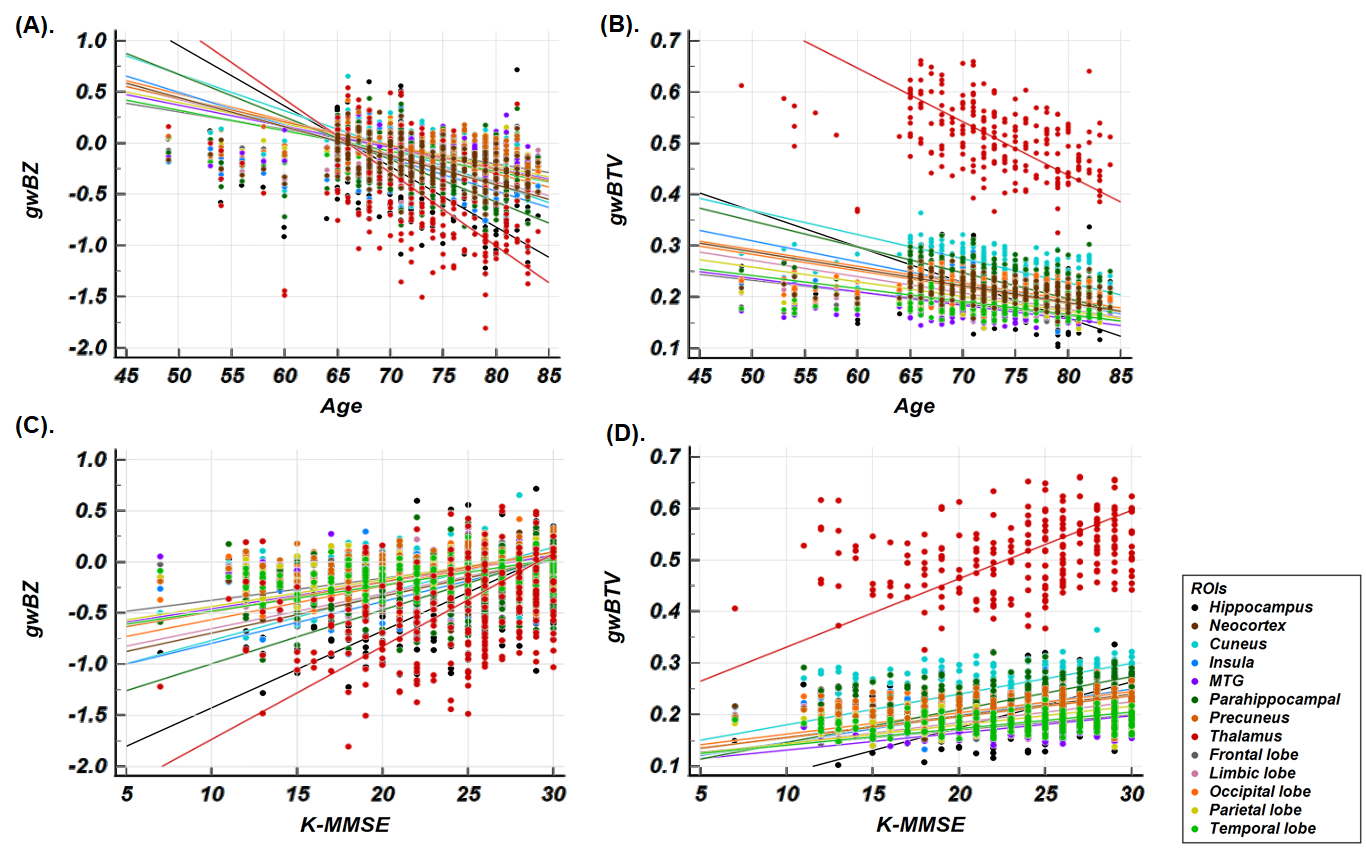


**Supplementary Figure S5. Graphical illustration of Table 3, which lists results of correlation analysis of gray-white matter boundary Z-score(gwBZ) and its tissue volume (gwBTV) with Korean version of the Mini-Mental State Examination(K-MMSE) and Age in all ROIs.**

(A) Correlation of gwBZ and Age, (B) Correlation of gwBTV and Age, (C) Correlation of residual of gwBZ and K-MMSE, and (D) Correlation of residual of gwBTV and K-MMSE

gwBZ, gray-white matter boundary Z-score; gwBTV, gray-white matter boundary tissue volume; K-MMSE, Korean version of the Mini-Mental State Examination; MTG, middle temporal gyrus.
